# Magnetic Fe_3_O_4_ Nanoparticles Modified Hydroxyapatite Whisker: A Novel Framework with Superior Osteogenic Efficacy

**DOI:** 10.1002/advs.202509715

**Published:** 2025-08-11

**Authors:** Xue Zhang, Qiang Wang, Wanxin Zheng, Zhijian Li, Lanfeng Qu, Yulou Tian, Dan Zhang, Tingting Yan, Qing Zhou

**Affiliations:** ^1^ Liaoning Provincial Key Laboratory of Oral Diseases School and Hospital of Stomatology China Medical University Shenyang 110001 China; ^2^ Affiliated Stomatological Hospital of Jinzhou Medical University Jinzhou 121000 China; ^3^ Faculty of Materials Science and Engineering Kunming University of Science and Technology Kunming 650000 China

**Keywords:** Hydroxyapatite whisker, Fe_3_O_4_ nanoparticles, magnetic field, mechanical transduction, osteogenic differentiation

## Abstract

The valid repair of bone defects requires considering precise elements mirroring native tissue and the efficiency of treatment reality. Magnetic biomaterial‐mediated mechanotransduction emerges as a critical mechanism for enhancing osteogenic differentiation and bone regeneration. Here, enhancement of osteogenic capacity is achieved in a hydroxyapatite whisker (HAw) by integrating with magnetic Fe_3_O_4_ nanoparticles (HAw/Fe_3_O_4_). The HAw/Fe_3_O_4_ exhibited excellent magnetic responsiveness and controllable alignment along magnetic field (MF) vectors enabled precise delivery of programmed mechanical stimuli. In vitro and in vivo experiments showed that Fe_3_O_4_ nanoparticles modified HAw has satisfactory biocompatibility and osteoinductivity, while the synergistic efficacy of magnetic biomaterial with external MF stimulation significantly accelerated bone matrix mineralization. The magnetic HAw/Fe_3_O_4_ with a MF significantly increased the expression of osteogenic markers and improved bone repair. The mechanosensitive ion channel of Piezo1 enabled efficient transcellular mechanotransduction through the MAPK pathway in response to the magnetic HAw/Fe_3_O_4_ with MF stimulation. The current study demonstrates that the HAw/Fe_3_O_4_ magnetic framework ‐ developed through Fe_3_O_4_ nanoparticle integration to enhance HAw osteoinductivity via coordinated MF application and mechanobiological modulation ‐ served as an optimal therapeutic platform for delivering programmed mechanical cues to activate Piezo1‐mediated mechanotransduction cascades, ultimately accelerating bone tissue regeneration.

## Graphical abstract

A novel magneto‐active bone regeneration framework was engineered by integrating Fe_3_O_4_ nanoparticles with hydroxyapatite whiskers (HAw/Fe_3_O_4_). This composite acquired magnetic responsivity, enabling programmable microstructural alignment and amplified mechanobiological cues under magnetic fields, significantly enhancing osteogenesis and bone regeneration to offer a paradigm‐shifting approach for spatiotemporal bone reconstruction.

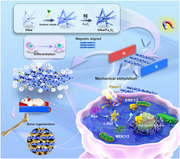

## 1 Introduction

Bone defects were primarily caused by surgical interventions, traumatic injuries, or neoplastic pathologies, and critical‐sized defects in osseous tissues exhibited limited intrinsic regenerative capacity post‐injury. The challenge of bone regeneration stemmed from the reconstruction of various biochemical compositions^[^ ^1^ ^]^ and physical structures in bone tissue. In bone tissue engineering (BTE), hydroxyapatite (HA) was widely used as a bone regeneration bio‐material or material‐based frame due to its similarity in native tissue composition.^[^ ^2^, ^3^, ^4^, ^5^ ^]^ Hydroxyapatite whisker (HAw),^[^ ^6^, ^7^, ^8^ ^]^ as a synthetic whisker shaped nanocrystalline hydroxyapatite mirroring the morphology of HA, was a calcium phosphate mineral used to ameliorate the bending and compressive properties of bioceramics,^[^ ^9^, ^10^ ^]^ as well as to repair bone tissue.^[^ ^11^, ^12^ ^]^ However, it was revealed that HAw exhibited insufficient osteoinductivity in vivo, which imposed restrictions on its clinic application.^[^ ^6^, ^7^ ^]^ To optimize osseointegration and bone defect repair, numerous modified HAw‐based biomaterials have been developed, which demonstrated exceptional biocompatibility, osteoconductivity, and antibacterial activity while maintaining intrinsic biological performance. Surface modification through the incorporation of osteoinductive components ‐ including nano oxide,^[^ ^13^ ^]^ polymer materials,^[^ ^14^ ^]^ metallic elements,^[^ ^15^ ^]^ and bioactive ions^[^ ^2^, ^16^ ^]^ ‐ have been shown to effectively enhance the osteoinductive potential of HAw substrates.

More recently, a noninvasive strategy of manufacturing a magnetic‐bioactive coating on material‐based matrixes controlled by an external MF to provide mechanical and physical cues for stem cells would highly effectively meet the clinical needs.^[^ ^17^, ^18^ ^]^ Several modalities of materials modification for mechanobiological interventions stirred encouraging possibilities to enhance cell survival, osteo‐differentiation, and bone regeneration.^[^ ^19^ ^]^ Mechanical signal stimulation, as the initial regulating link of stem cell differentiation, plays vital roles in bone regeneration^[^ ^20^, ^21^ ^]^; of these mechanical signal interventions, the magnetic‐responsive biomaterials as a convenient method to orchestrate cell fate, have been tentatively used for regulating cell‐material mutual effects.^[^ ^22^ ^]^ Iron oxide NPs (IONPs), as magnetic nanoparticles (MNPs) can be manipulated by remote MF, have been widely detected for their potential opportunities of surface modification in nanomedicine.^[^ ^23^ ^]^ The IONPs commonly referred to the magnetic bio‐materials, such as magnetite (Fe_3_O_4_),^[^ ^13^ ^]^ that were widely used for its excellent magnetization and minimum toxicity in the nanomagnetism.^[^ ^24^ ^]^ It was reported that Fe_3_O_4_ significantly increased the osteogenic differentiation of MC3T3 osteoblasts in a MF.^[^ ^25^ ^]^ Besides, nanocomposites via integrating Fe_3_O_4_ with scaffolds possessed comprehensive properties for biomedical tissue engineering applications.^[^ ^26^ ^]^ The grafting Fe_3_O_4_ was based on its superparamagnetic capacity, fast responding to the environment MF, and particularly the ability of enhancing mechanical stimulation to accelerate bone regeneration. Fe_3_O_4_/MC coatings on the titanium substrate demonstrated enhancement of osteogenic differentiation in the MC3T3‐E1 cells, especially the positive effect amplified under an external MF.^[^ ^18^ ^]^ Furthermore, the MC coatings with different distributions of the magnetic nanoparticles revealed mechanical stimulation for osteogenesis enhancement with exposure to a static magnetic field (SMF). Notably, the past few decades have witnessed potential development of MF stimulation^[^ ^27^ ^]^ and magnetic‐responsive biomaterials as the molecular force transducer for regulating in the molecular and cellular processes of triggering cell surface receptors, remodeling the cytoskeleton, and activating cell differentiation.^[^ ^20^, ^28^ ^]^

Cells can exquisitely sense and respond to external biochemical and mechanical stimulation, that are triggered by biological material or the external micro‐environment. Mechanical transduction from extracellular to intracellular may occur any process or position between cells and microenvironment or between the mechanical receptors of a single cell with diverse factors for the regulation of bone homeostasis and remodeling.^[^ ^13^ ^]^ The transfer of mechanical stimuli to regulate intracellular biochemical signaling pathways depends on mechanical stimuli receptors on the cell, which could be ion channels, integrins, gap junction proteins, ECM, cellular skeletal ingredients and primary cilia.^[^ ^18^, ^29^, ^30^ ^]^ Mechanosensitive Piezo1 is an ion channel protein discovered in 2010, that has a trimeric propeller‐shaped structure, including three blades, a central cap, and core transmembrane segments.^[^ ^31^, ^32^ ^]^ Moreover, Piezo1 is a nonselective Ca^2+^‐permeable cation channel and regarded as a crucial sensor for mechanical stimuli signals. Stem cells can rapidly respond to mechanical stimulation by Piezo1, which is localized at or near the plasma membrane and converts mechanical stimuli through Ca^2+^ response from the extracellular environment into intracellular biological signals, regulating various physiological processes through cell signal transduction pathways and finally modulating bone homeostasis and bone regeneration.^[^ ^33^, ^34^ ^]^ During the process of mechanical transduction, signal pathways (integrin, MAPK, BMP, ROCK, WNT, and NF‐𝜅B) are activated within cells under mechanical stimulation.^[^ ^13^, ^35^ ^]^ It was reported that the enhancement of osteogenic differentiation on the stimulated MC3T3‐E1 cells in an MF relied on an augmentative mechanotransduction signaling pathway.^[^ ^18^ ^]^ The downstream osteogenic‐related factors of these signaling pathways then upregulated the expression of the genes, including ALP, OCN, Col‐I, and Runx2.^[^ ^26^ ^]^

**Figure 1:**
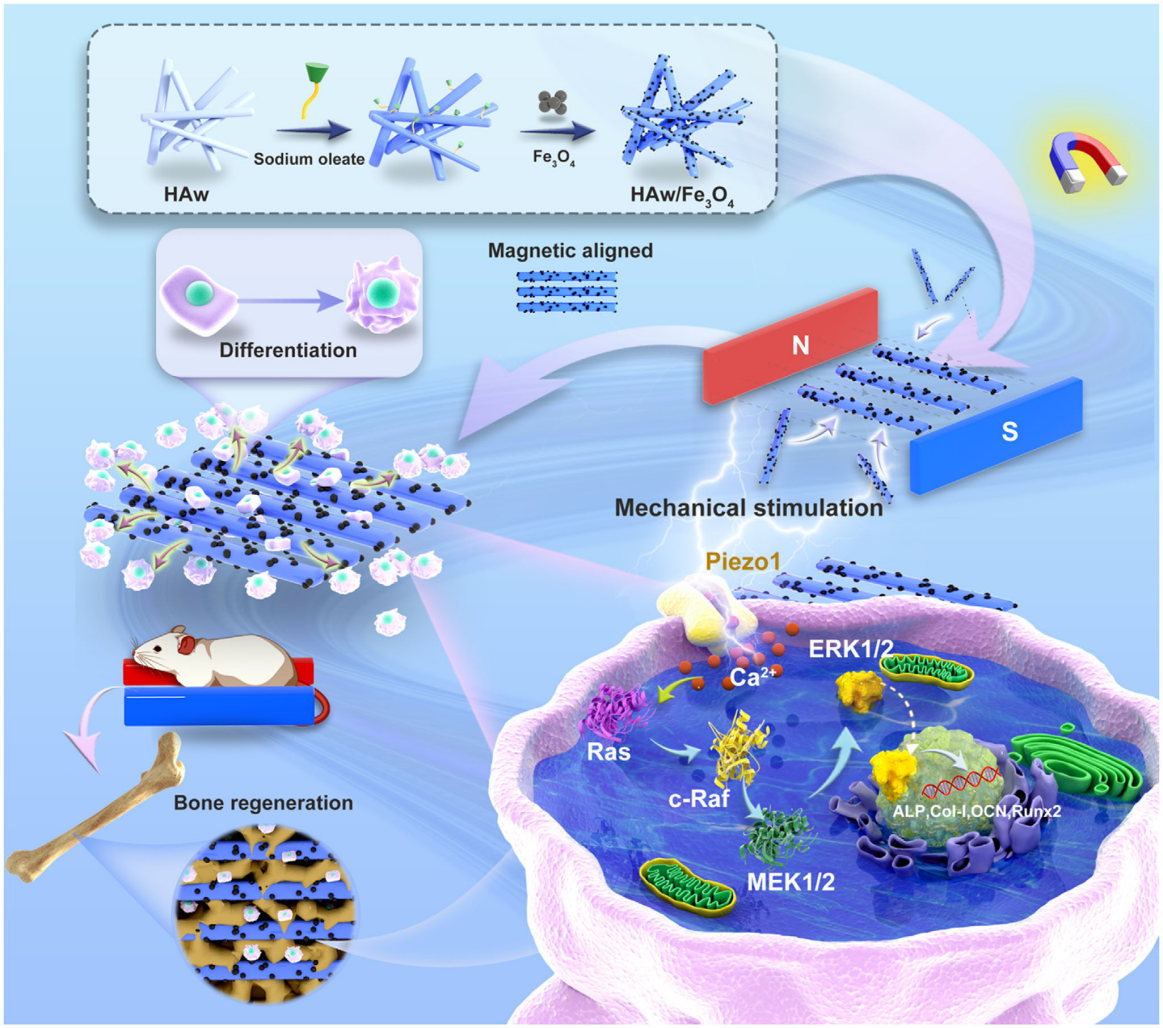
The design and application of the magnetic HAw/Fe_3_O_4_ for bone differentiation and reconstruction. A schematic illustration showed that the composite magnetic biomaterials were assembled through modified hydroxyapatite whiskers with Fe_3_O_4_ nanoparticles. This novel magnetic framework achieved an ideal enhanced osteogenic differentiation and bone regeneration via the magnetic HAw/Fe_3_O_4_ in a MF.

Herein, the objective of this work was to develop a novel framework possessed physicochemical property of HAw and magnetism, i.e. magnetic Fe_3_O_4_ nanoparticles modified HAw (HAw/Fe_3_O_4_). The valid tactics of the novel framework were attempted to preserve the reinforcing and toughening effects of raw HAw^[^ ^15^, ^36^ ^]^ and represented a facile approach to improve the osteoinductivity and cell adaptability, as well as highly sensitivity to an MF as mechanical and physical cues for stem cells in tissue regeneration (**Figure** **1**). Although the effects of Fe_3_O_4_ and MF stimulation were evident, the molecular mechanism of osteogenic differentiation in stem cells induced by Fe_3_O_4_‐based tissue engineering materials had not been fully elucidated. It was of great interest to explore how the mechanotransduction in stem cells actuation by HAw/Fe_3_O_4_. We hypothesized that bone marrow mesenchymal stem cells (BMSCs) sensed mechanical stimuli through Piezo1, which served as critical mechanotransduction components mediating the conversion of magnetically induced mechanical signals from HAw/Fe_3_O_4_ composites under MF exposure into downstream signaling pathway activation (Figure 1).

In view of the above statements, we thus investigated whether magnetic HAw/Fe_3_O_4_ in an MF could enhance the osteogenic capacity both in vitro and in vivo, as well as the role of Piezo1 was also evaluated in the processes of mechanical transduction. As a convenient approach to manipulate cell behaviors in MF, the novel framework possesses a promising prospect for superior osteogenic efficacy to optimize bone repair, which presumably provides a new strategy for bone defect healing efficiently and precisely.

## 2 Results and Discussion

### 2.1 Fabrication and Characterization of Magnetic HAw/Fe_3_O_4_

**Figure 2:**
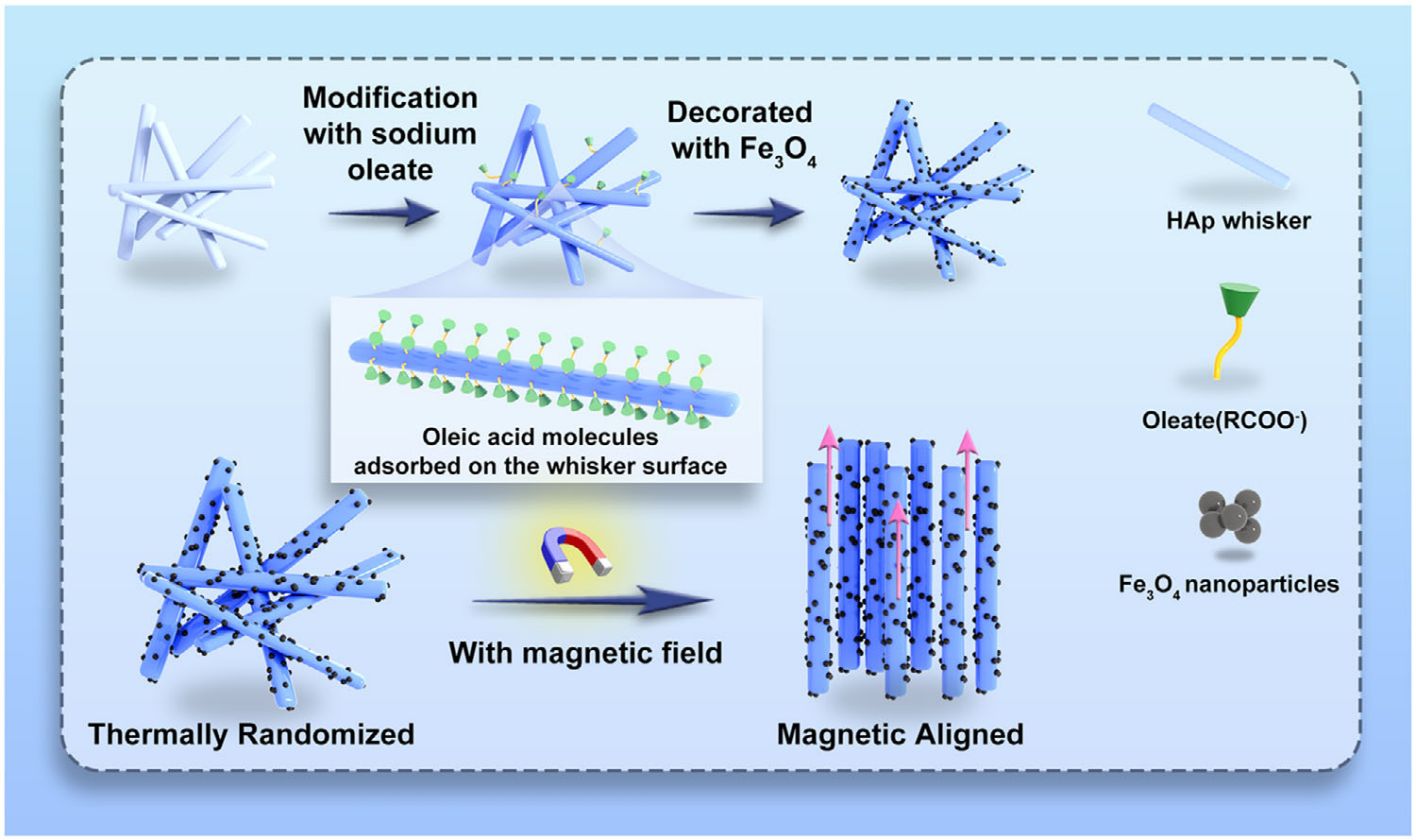
The schematic fabrication process for the magnetic HAw/Fe_3_O_4_.

**Figure 3:**
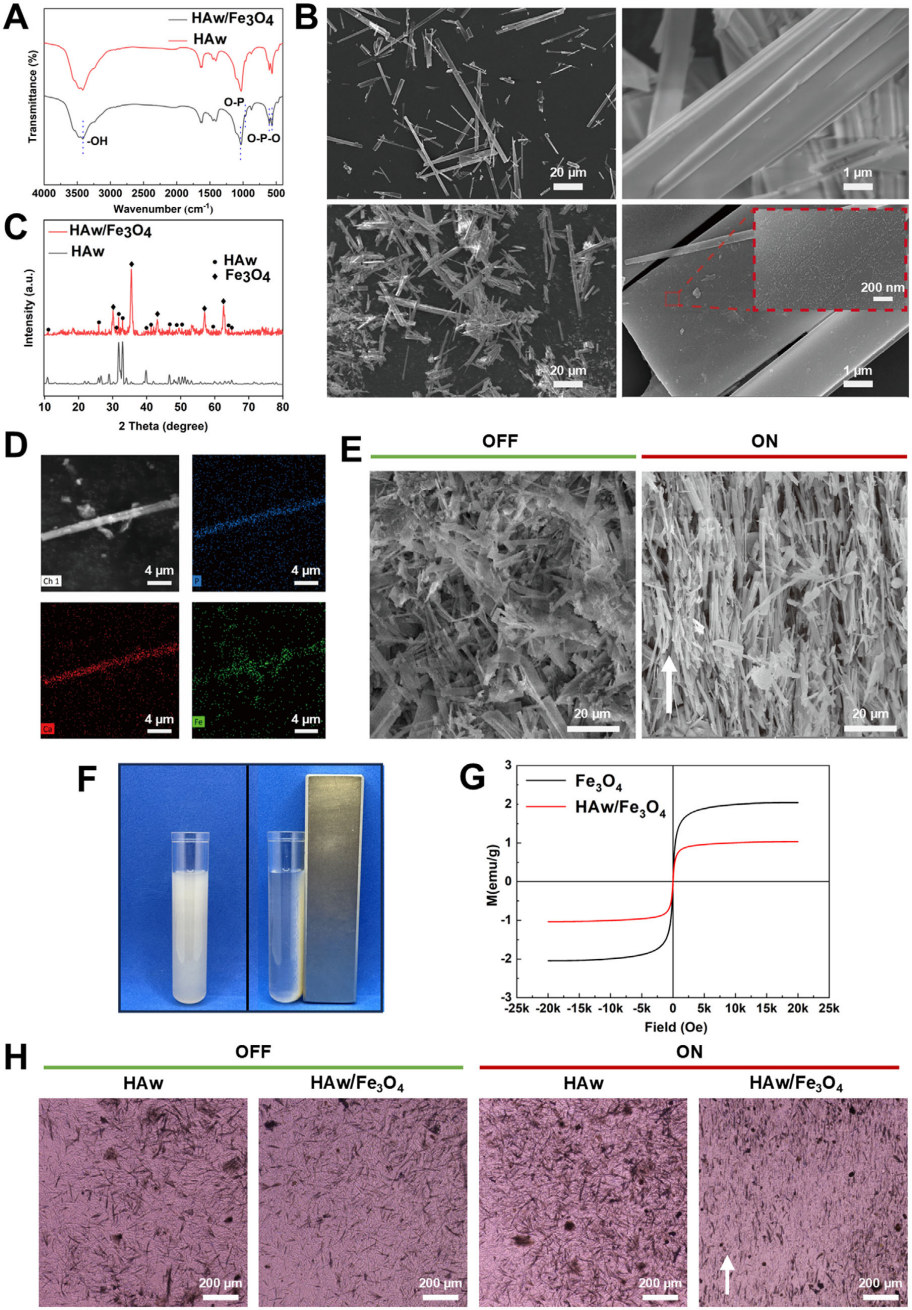
Fabrication and characterization of the magnetic HAw/Fe_3_O_4_. A) FTIR spectra of the HAw and HAw/Fe_3_O_4_. B) The microstructure revealed in SEM, (a, b) raw HAw (c, d) the magnetic HAw/Fe_3_O_4_. C) X‐ray diffraction (XRD) for raw HAw and the magnetic HAw/Fe_3_O_4_. D) Elemental analysis by EDS on the surface of magnetic HAw/Fe_3_O_4_. E) The arrangement of HAw/Fe_3_O_4_ under different patterns of the MF (“OFF” and “ON”). “OFF”, without an MF; “ON”, with an MF. F) Photos showed the process of the magnetic HAw/Fe_3_O_4_ magnetization. G) Magnetization curves of the magnetic HAw/Fe_3_O_4_ measured by VSM. (H) Images observed by the light microscope with different patterns of the MF (“OFF” and “ON”). The white arrow indicated an aligned parallel distribution of the magnetic HAw/Fe_3_O_4_ in an MF.

HAws were synthesized by the hydrothermal homogeneous precipitation method described above,^[^ ^2^, ^37^ ^]^ and used as the base material. To achieve a parallel distribution simulating natural tissues and provide mechanical stimulation to drive cellular mechanotransduction,^[^ ^38^ ^]^ we designed and fabricated a magnetic nanocomposite by integrating HAw with Fe_3_O_4_ nanoparticles in this study (Figure 1 and **Figure** **2**). Magnetic HAw/Fe_3_O_4_ was successfully synthesized by loading nano‐Fe_3_O_4_ particles (10 wt.%) onto HAw. Fourier transform infrared spectroscopy (FTIR) confirmed characteristic diffraction peaks at 3415, 1029, and 564 cm^−1^, representing PO_4_^3‐^ and OH^‐^ (**Figure** **3**A). The characteristic peak of Fe‐O at 551 cm^−1^ overlapped with the absorption peak of PO_4_^3‐^, making it difficult to distinguish from the absorption peak of HAw. In the XRD pattern of Figure 3C, the characteristic diffraction peaks of HAw/Fe_3_O_4_ at 2θ = 10.820, 31.773, 32.196, and 39.818 belonged to HAw, while those at 2θ = 29.916, 35.371, 43.138, and 62.517 were characteristic diffraction peaks of Fe_3_O_4_. Quantitative phase composition analysis by the reference intensity ratio (RIR) method showed that the content of HAw was 91.3% and that of Fe_3_O_4_ was 8.7% (Table S3, Supporting Information), further illustrating the successful synthesis of magnetic HAw/Fe_3_O_4_. Statistical analysis of the FWHM of the main crystal planes of HAw showed an increase in the grain size of the main crystal planes (Table S1, Supporting Information), which may be due to the surface of HAw being covered by Fe_3_O_4_ nanoparticles (B‐(d)). The diffraction of HAp crystals was weakened. As shown in Figure 3B, the whisker length of HAw decreased, the width increased, and it became short and thick after loading Fe_3_O_4_ particles, which was confirmed by the smaller FWHM and larger grain size of the (100) and (112) crystal planes. In Figures 3B‐(c) and B‐(d), the surface of HAw was smooth, while the surface of HAw/Fe_3_O_4_ had a Fe_3_O_4_ coating film with an average particle size of ≈5–10 nm. Table S2 and Figure S1 (Supporting Information) showed that the crystallinity of HAw and HAw/Fe_3_O_4_ did not change. The EDS analysis of HAw/Fe_3_O_4_ in Figure 3D further confirmed the presence of surface Fe_3_O_4_ and that the preparation of magnetic HAw/Fe_3_O_4_ by this method had no effect on the crystallinity of HAw. In Figure 3F, it can be clearly seen that the magnetic HAw/Fe_3_O_4_ was significantly manipulated to move toward the MF direction. In the SEM image (Figure 3E), HAw/Fe_3_O_4_ was arranged disorderly without an MF, and after applying an external MF, HAw/Fe_3_O_4_ was arranged along the direction of the MF. In Figure S2 (Supporting Information), the angle between HAw/Fe_3_O_4_ and the horizontal direction under N‐MF (NO magnetic field) and MF conditions was significantly observed, and it approached vertical under the magnetic field condition (*p* < 0.0001). The magnetic hysteresis loops of Fe_3_O_4_ and HAw/Fe_3_O_4_ were tested by VSM. It can be preliminarily seen in Figure 3G that both curves were reversible S lines. The saturation magnetization of magnetite was 2.037 emu g^−1^, and that of HAw/Fe_3_O_4_ was 1.035 emu g^−1^. The saturation magnetization of HAw/Fe_3_O_4_ was smaller than that of magnetite (Figures S5, Supporting Information), which was because a small part of magnetite particles was on HAw/Fe_3_O_4_, and the remanent magnetization and coercivity were small (Figures S3 and S4, Supporting Information). Therefore, it can be explained that both materials have the magnetic properties of magnetite and were easy to magnetize and demagnetize. The results of raw HAw and magnetic HAw/Fe_3_O_4_ with or without MF using light microscope showed that HAw exhibited a disordered state whether in an MF or not. When without MF stimulation involved, it was observed that a random arrangement on the group of magnetic HAw/Fe_3_O_4_ in the “OFF” state. Whereas unlike HAw, the magnetic HAw/Fe_3_O_4_ in the “ON” state displayed a narrow orientation aligned parallel distribution along the direction of the MF with magnetic control applied (Figure 3H). Through the above successful preparation of magnetic HAw/Fe_3_O_4_ and the confirmation of its magnetic effect, using biomaterial‐mediated in situ physical cues to achieve mechanotransduction potentially serves as a method to manipulate stem cell differentiation and stimulate bone regeneration.^[^ ^39^ ^]^ The next work will study that excellent magnetism can activate mechanotransduction by transmitting mechanical stimulation through magnetic HAw/Fe_3_O_4_, thereby coordinating cell fate.

### 2.2 Gradient Adaptable Concentration Magnetic HAw/Fe_3_O_4_ for BMSCs

**Figure 4:**
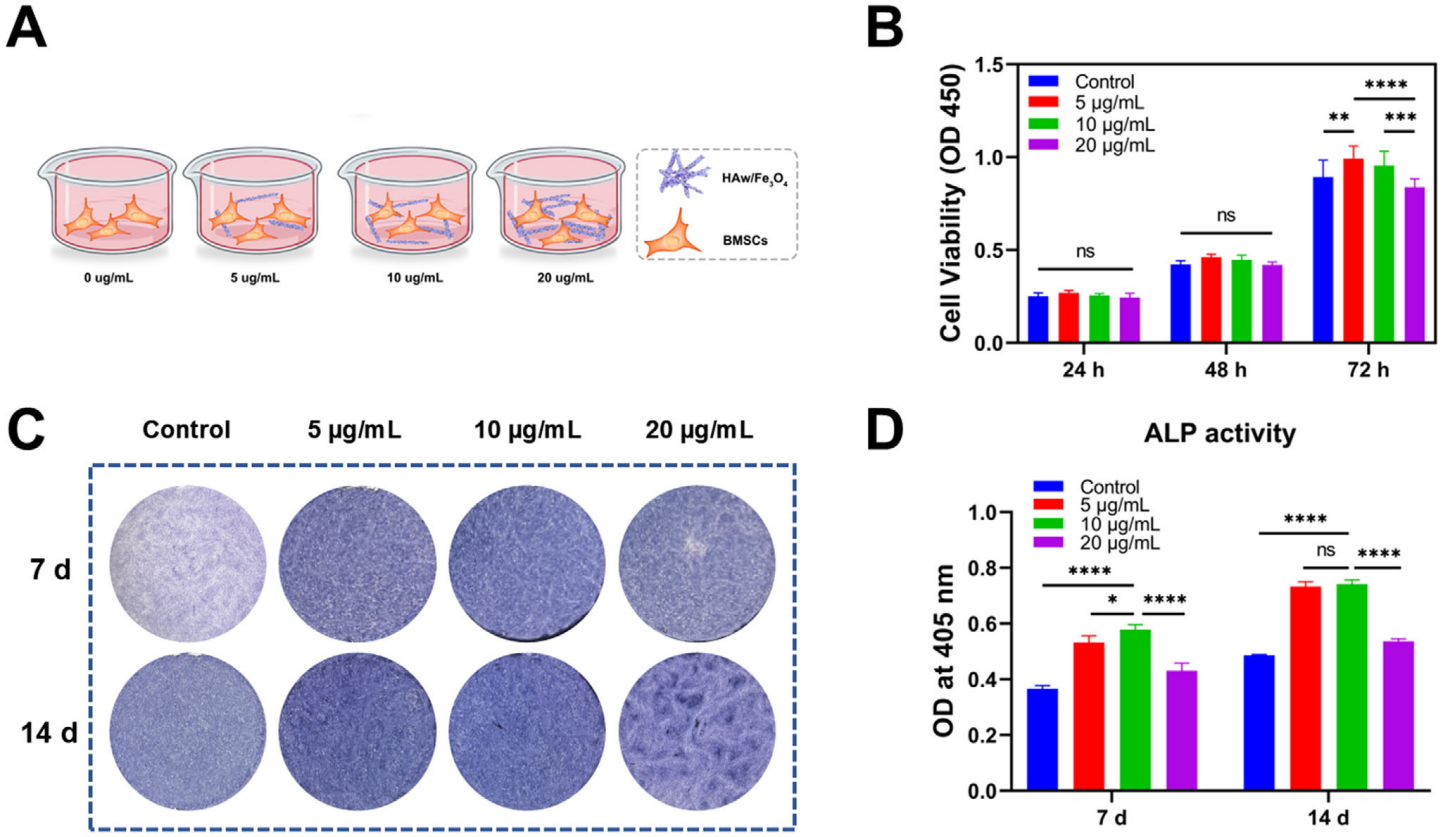
Gradient adaptable concentrations of magnetic HAw/Fe_3_O_4_ for BMSCs. A) Strategy diagram for BMSCs co‐cultured in the gradient adaptable concentrations of magnetic HAw/Fe_3_O_4_ (0, 5, 10, and 20 µg mL^−1^). B) Cell viability of BMSCs co‐cultured with gradient concentrations of magnetic HAw/Fe_3_O_4_ for 24, 48, and 72 h (*n* = 5). C)ALP staining and D) quantification of ALP activity for 7 and 14 days (*n* = 3). ^*^ *p* < 0.05; ^**^ *p* < 0.01, ^***^ *p* < 0.001, ^****^ *p* < 0.0001, and ns, no significance.

Biocompatibility assay and osteogenic property were of great importance to evaluate biomaterials for bone repair, and the optional surroundings of cell survival were vital to evaluate the magnetic materials in vitro. Considering independent effect of the magnetic HAw/Fe_3_O_4_ without the MF on BMSCs, the different gradients of the magnetic HAw/Fe_3_O_4_ co‐cultured with BMSCs were detected (**Figure** **4**A). CCK‐8 and ALP assays were involved to determine the optional co‐cultured concentration of the magnetic HAw/Fe_3_O_4_ without MF intervention. The CCK‐8 assay showed that the optical density (OD) value of cells on the magnetic HAw/Fe_3_O_4_ in each group increased significantly with the prolongation of the culture time, revealing that magnetic HAw/Fe_3_O_4_ with gradient adaptable concentration showed perfect cell biocompatibility (Figure 4B). With the decrease of concentration, the cell proliferation rate of the samples increased. Significantly, the OD value of cells on the magnetic HAw/Fe_3_O_4_ (5, 10 µg mL^−1^) groups increased compared with the control group and the group of 20 µg mL^−1^ HAw/Fe_3_O_4_. In other word, lower concentration (5 and 10 µg mL^−1^) promoted cell proliferation compared with the control group, but in fact higher concentration (20 µg mL^−1^) was unable to facilitate effect. To further notarize the co‐cultured concentration of magnetic HAw/Fe_3_O_4_, the osteoinductivity of BMSCs on gradient concentration HAw/Fe_3_O_4_ was implemented. The alkaline phosphatase (ALP) staining showed that the osteoinductivity of the magnetic HAw/Fe_3_O_4_ groups were superior to that in control groups at 7 and 14 days, and the effect in the lower concentration HAw/Fe_3_O_4_ (5, 10 µg mL^−1^) preceded that in the group of 20 µg mL^−1^ HAw/Fe_3_O_4_ (Figure 4C). Moreover, after BMSCs seeded on the magnetic HAw/Fe_3_O_4_ for 7 days, quantitative value of ALP in the moderate group (10 µg mL^−1^) was significantly higher than that in other groups (5 and 20 µg mL^−1^), as showed in Figure 4D. At 14 days, there was no significantly difference between 5 and 10 µg mL^−1^ groups, whereas significantly higher than higher concentration group (20 µg mL^−1^). Thus, the group of magnetic HAw/Fe_3_O_4_ (10 µg mL^−1^) exhibited ideal biocompatibility and osteogenic inductivity, applied as the optimal choice for follow‐up experiments.

### 2.3 Magnetic HAw/Fe_3_O_4_ in Suitable MF

**Figure 5:**
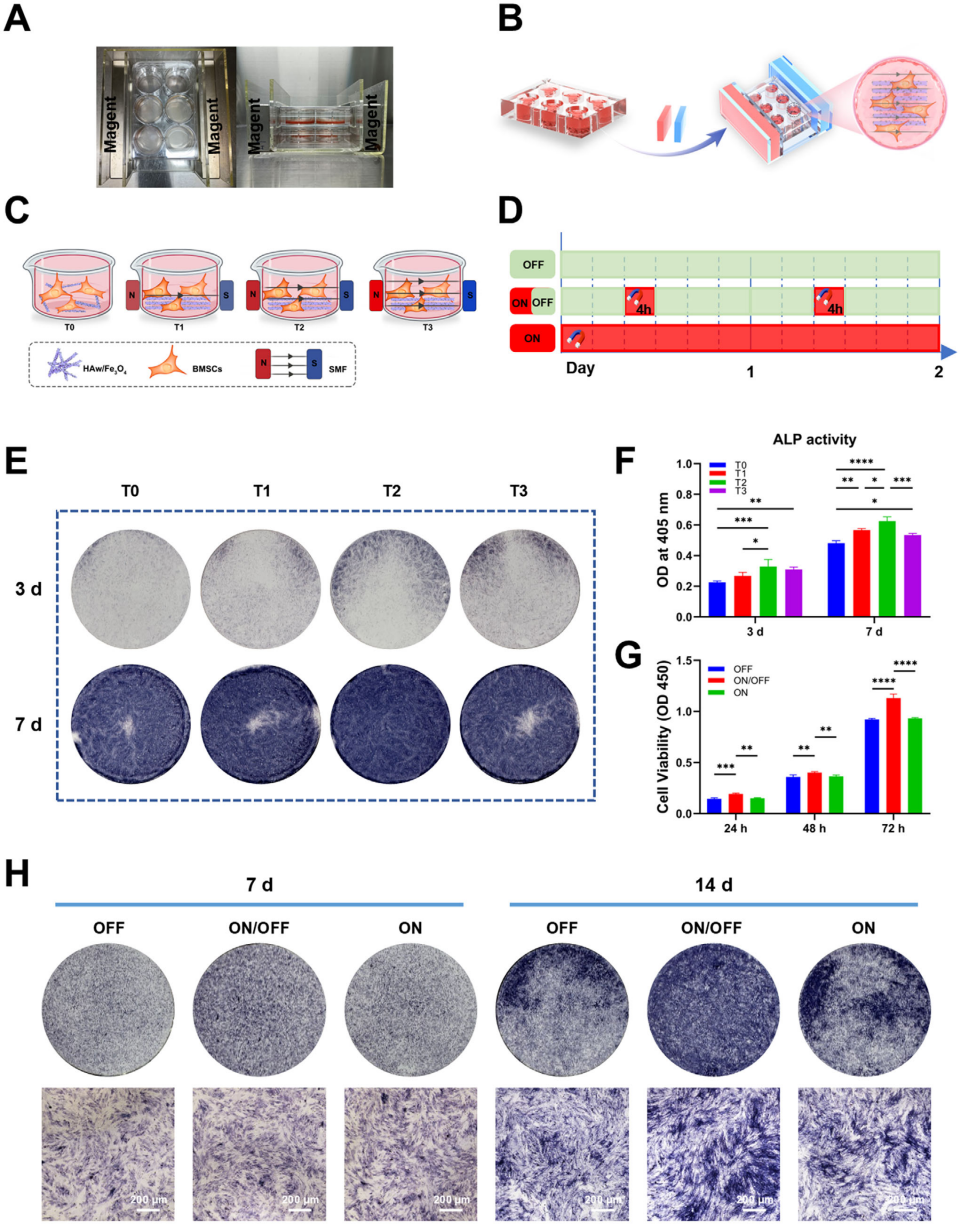
MF stimulation for BMSCs co‐cultured in the magnetic HAw/Fe_3_O_4_. A) Photographs and B) Strategy diagrams of the magnetic device. C) Diagram for BMSCs co‐cultured in the magnetic HAw/Fe_3_O_4_ with the gradient strengths of MF (T0, T1, T2, and T3). D) Time axes of BMSCs co‐cultured in the magnetic HAw/Fe_3_O_4_ under the “OFF”, “ON” (24 h/day), or “ON/ OFF” (4 h/day) state. E) ALP staining and F) quantification of ALP activity in the gradient strength MFs for 3 and 7 days (*n* = 3). G) Cell viability of BMSCs co‐cultured via different MF patterns (“OFF” “ON”, and “ON/ OFF”) for 24, 48, and 72 h (*n* = 4). (H) ALP activity of BMSCs with different MF patterns for 7 and 14 days. ^*^ *p* < 0.05; ^**^ *p* < 0.01, ^***^ *p* < 0.001, and ^****^ *p* < 0.0001.

The MF was also a critical factor for cells co‐cultured on the magnetic HAw/Fe_3_O_4_ and played a vital role in the process of bone tissue regeneration by affecting stem cell fate to enhance the expression of osteoblastic phenotype.^[^ ^40^, ^41^ ^]^ Nevertheless, the influence of MF parameters on the physiological function of BMSCs was complex, leading to the MF intensity and application time needed to be fully elucidated. Herein, the loading protocols of MF on BMSCs had been determined based on a series of screenings after evaluating cell proliferation and osteogenic differentiation of BMSCs. To explore the optimal MF stimulation intensity and frequency on BMSCs, we used a designed magnetic device (**Figure** **5**A, B) with different intensity (150, 270, and 350 mT) and adjusted MF application time with intermittent or continuous stimulation (Figure 5C, D) to determine the optimal MF stimulation application for the follow‐up tests. The results revealed that the T2 group (270 mT) exhibited significantly higher ALP activity compared to both the T0 group and other experimental samples (T1 and T3), as illustrated in Figure 4E,F. ALP activity displayed optimal osteoinductivity in the T2 group, consistent with the results of already reported studies.^[^ ^28^, ^35^ ^]^ Various MF strengths possessed different effects on cell behaviors. Static magnetic field (SMF) was classified as a weak (< 1 mT), moderate (1 mT to 1 T), strong (1T to 5 T) and ultrastrong (> 5 T) field, which had been accepted in the scientific community.^[^ ^40^ ^]^ In preclinical studies, medium strength MF stimulation was the widely used to activate magnetic scaffolds for biological research, and was confirmed that promoting cell proliferation, deposition of the mineralized matrix, and osteogenesis of cells.^[^ ^42^, ^43^ ^]^ Under moderate‐intensity SMF exposure (280 mT), the magnetic‐responsive hydrogel induced phenotypic switching of encapsulated macrophages from M1 to M2, which subsequently promoted osteogenic differentiation and enhanced immunomodulation‐mediated bone regeneration.^[^ ^35^ ^]^ The moderate intensity SMFs could influence the adipo‐osteogenic differentiation, and SMFs ranging from 0.2 to 0.6 T markedly alleviated bone mass loss in an intensity‐dependent manner.^[^ ^44^ ^]^ In addition to the MF intensity, the action time was another important parameter affecting the cellular metabolism by the SMF. SMF‐driven in situ magnetic interventions demonstrated programmable temporal regulation capabilities, allowing time‐scheduled manipulation of osteogenic differentiation during bone repair.^[^ ^35^ ^]^ Comparative analysis using CCK‐8 and ALP assays was conducted under 270 mT MF exposure with distinct stimulation regimens (4 vs. 24 h day^−1^), revealing time‐dependent cellular responses (Figure 4G,H). BMSCs co‐cultured under MF device in the “ON/OFF” state (4h/day) exhibited a significantly higher cell viability and efficiently osteoinductivity compared to other groups, which indicated that intermittent stimulus applications of the MF performed a high efficiency to manipulate cell osteogenic differentiation. In previous study of magnetic biomaterials, the tensile stress status of the fibers in extracellular matrix was implied by evaluating the interaction of the materials and cells in response to the magnetic actuation direction,^[^ ^45^ ^]^ revealing that tensile stress was a vital key for mechanical stimulation to regulate cell behavior. Thus, it was assumed that high efficiency of intermittent stimulus might be due to the tensile stress status derived from the deformation frequency of the magnetic HAw/Fe_3_O_4_ in an MF and then amplified mechanical stimulation in situ to impress on adherent cells.

### 2.4 Magnetic HAw/Fe_3_O_4_ Under MF Stimulation on Cell Morphology

**Figure 6:**
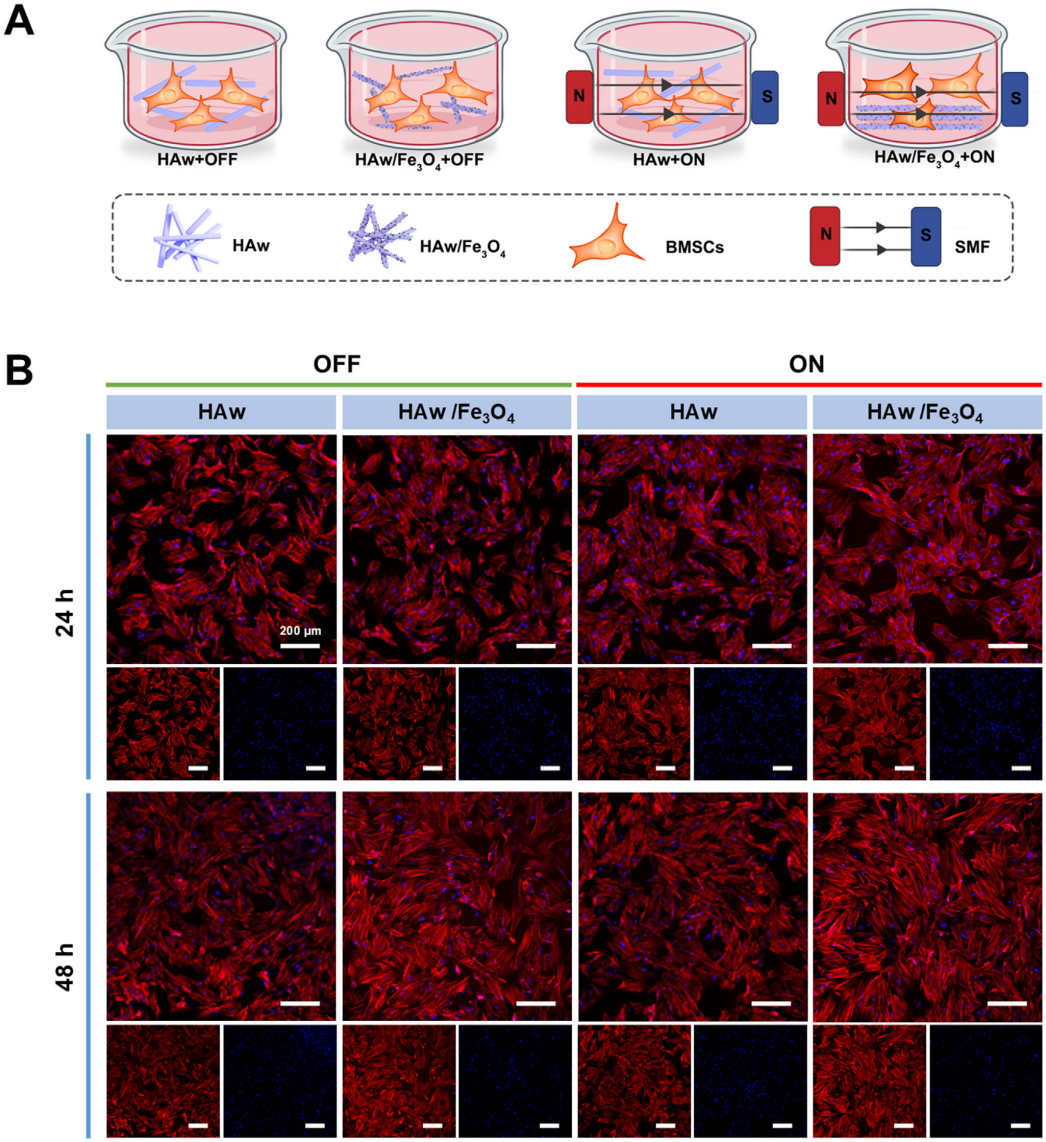
Cell morphology of BMSCs co‐cultured with HAw and the magnetic HAw/Fe_3_O_4_ in the MF. A) Strategy diagrams for BMSCs co‐cultured under the “OFF” and “ON” (4 h day^−1^) state. B) Images of fluorescein‐labeled phalloidin on cells co‐cultured with HAw and the magnetic HAw/Fe_3_O_4_ by different MF patterns (“OFF” and “ON”).

Biocompatibility, such as cell morphology and proliferation played a crucial role to assess bio‐materials in BTE. Since pre‐cultivation conditions screened in a designed magnetic device, we next carried out a series of comparison evaluations in the case of co‐cultured BMSCs on the pure HAw and magnetic HAw/Fe_3_O_4_. To distinguish the impact of MF or magnetic material on osteogenic differentiation of BMSCs, pure HAw treated with MF stimulation and magnetic HAw/Fe_3_O_4_ without an external MF were parallel analyzed with pure HAw in the “OFF” state and magnetic HAw/Fe_3_O_4_ in the “ON” state (**Figure** **6**A). In the study, BMSCs consistently exhibited characteristic fibroblast‐like morphology in all experimental groups, with well‐spread cellular architecture observed on all substrate surfaces after 24 and 48 h of culture (Figure 6B). Compared to pure HAw substrates, BMSCs cultured on the magnetic HAw/Fe_3_O_4_ composite demonstrated enhanced cellular spreading and a significantly larger cell‐covered area, indicating that the incorporation of Fe_3_O_4_ nanoparticles improved cell adhesion capacity. Notably, extracellular or temporary matrix molecules including fibronectin, vitronectin, collagen, laminin or fibrin participated cell attachment in the adherence interface between cells and biomaterials. Moreover, the physical and chemical properties of materials were vital to regulate cell fate involving cell adhesion and morphological variation. We envision that the magnetic HAw/Fe_3_O_4_ has good surface roughness via integrating Fe_3_O_4_ nanoparticles to provide more anchorage points for cytopseudopod. With the assist of MF, the magnetic HAw/Fe_3_O_4_ possessed a suitable micro‐environment with  an inerratic configuration for cell growth and attachment. Furthermore, the actin fibers of the cells on magnetic HAw/Fe_3_O_4_ had a tendency mostly oriented along the major axis of the MF stimulation, considering that the well‐arranged configuration could regulate cell morphology.

### 2.5 Magnetic HAw/Fe_3_O_4_ in an MF on cell proliferation and differentiation

**Figure 7:**
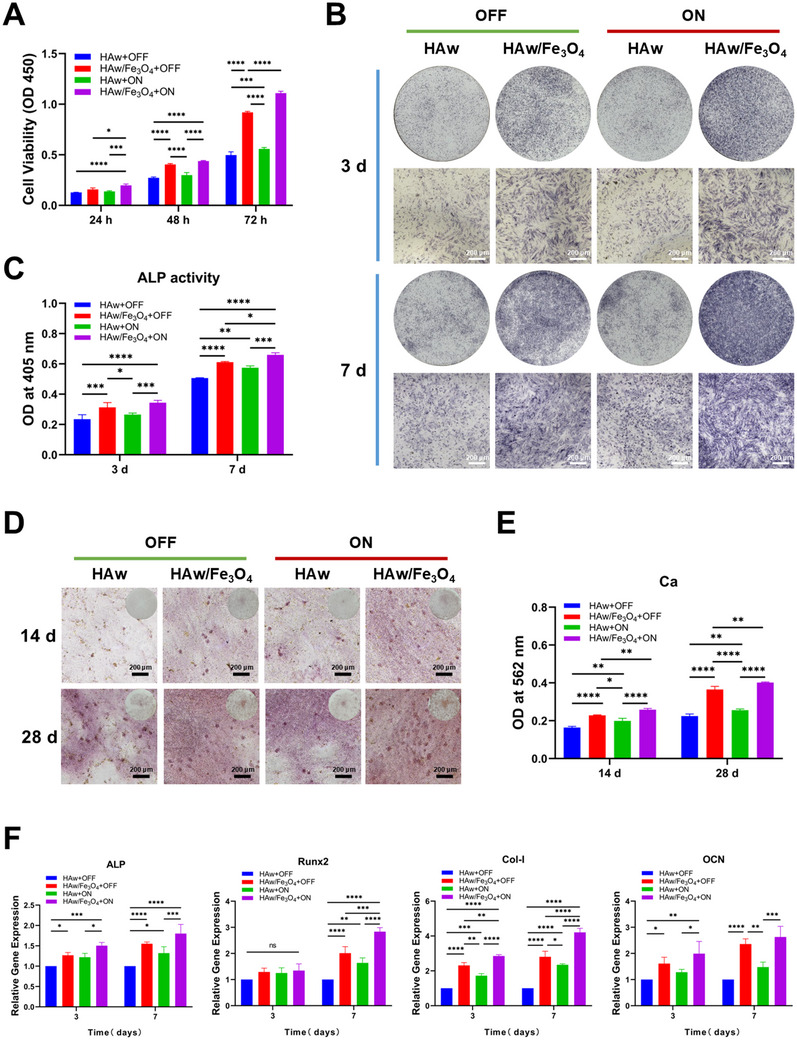
Proliferation and differentiation of BMSCs co‐cultured with HAw and the magnetic HAw/Fe_3_O_4_ in an MF. A) Cell viability of BMSCs co‐cultured via different MF patterns (“OFF” and “ON”) for 24, 48, and 72 h. B) ALP staining and C) quantification of ALP activity by different MF patterns for 3 and 7 days. D) Alizarin red staining and E) mineralized nodule quantification of BMSCs co‐cultured via different MF patterns for 14 and 28 days. F) Relative mRNA expression levels of osteogenesis‐related genes (ALP, Runx2, Col‐I, and OCN) in BMSCs co‐cultured via different MF patterns. ^*^ *p* < 0.05; ^**^ *p* < 0.01, ^***^ *p* < 0.001, ^****^ *p* < 0.0001, and ns, no significance, *n* = 3.

After conducting the impact of magnetic HAw/Fe_3_O_4_ on cell morphology, experiments about cell proliferation and the osteogenic differentiation property of BMSCs treated with MF‐stimulated magnetic HAw/Fe_3_O_4_ were further analyzed. The CCK‐8 assay revealed that the magnetic HAw/Fe_3_O_4_ exhibited a significantly higher cell viability, compared to parallel groups of pure HAw. Under MF stimulation, the magnetic HAw/Fe_3_O_4_ composite group demonstrated the greatest enhancement. Furthermore, cells cultured on pure HAw substrates in the “ON” state exhibited a significantly higher OD value compared to those in the “OFF” state at the 72‐h time point (**Figure** **7**A). BMSCs proliferation was accelerated by the synergistic effect of the magnetic HAw/Fe_3_O_4_ with MF stimulation, suggesting that excellent biocompatibility and cell proliferation in the presence of the magnetic HAw/Fe_3_O_4_ and MF. ALP was denoted for osteogenic capacity using staining and quantitative measurement. BMSCs cultured with conditioned medium from the magnetic HAw/Fe_3_O_4_ groups exhibited stronger ALP staining than those of HAw groups (Figure 7B). In addition, the magnetic HAw/Fe_3_O_4_ group in the “ON” state exhibited intensified ALP staining compared with that without MF. Similar results were also verified in ALP quantitative measurement (Figure 7C). MF administration enhanced the ALP activity of the magnetic HAw/Fe_3_O_4_ comparing to parallel groups at 7 days. Moreover, a distinct difference was also observed between HAw groups with and without MF at 7 days, noticing the promotive effect of MF on osteogenic differentiation of BMSCs.

Next, matrix mineralization, as an indicator of osteogenic differentiation in the late stage, was evaluated using staining calcium nodules by Alizarin Red staining (ARS). Calcium mineral deposition revealed the stronger ARS results in the magnetic HAw/Fe_3_O_4_ groups than HAw groups both in the “ON” state and in the “OFF” state (Figure 7D). In addition, statistically significant differences were denoted between every two group comparisons by quantitative assessment of ARS (Figure 7E). Importantly, greater calcium deposition levels were discovered in the HAw/Fe_3_O_4_ groups than those of the HAw groups. Consistent with the ALP analysis, the magnetic HAw/Fe_3_O_4_ group in the “ON” state exhibited the best mineral deposition performance by staining and quantitative measurement of matrix mineralization, indicating that BMSCs on the magnetic HAw/Fe_3_O_4_ under MF stimulation secreted significant extracellular calcium deposits.

As the hallmarks of osteogenic differentiation in osteoblastic differentiation and bone mineralization, the target genes were then analyzed by RT‐PCR analysis. Compared to pure HAw groups, the levels of either early or late osteogenic markers were significantly up‐regulated in the magnetic HAw/Fe_3_O_4_ groups including ALP, Runx2, Col‐I, and OCN at 7days (Figure 7F). No obvious difference in the gene expression levels of Runx2 was observed in all the groups at 3 days, whereas the statistically significant differences were obvious among groups when MF stimulation and different materials imposed at 7 days. Moreover, the gene expression levels of ALP (HAw+OFF vs. HAw+ON at 7 days) and Col‐I (at 3 and 7 days) were significantly increased under MF stimulation contrast to the parallel control without MF. The magnetic HAw/Fe_3_O_4_ in the “ON” state exhibited the best osteogenesis, revealing that HAw/Fe_3_O_4_ with MF stimulation could effectively enhance the osteogenic responses and mineralization of BMSCs. Interestingly, the results involving the single efficiency of materials or MF on cell behaviors showed that the magnetic HAw/Fe_3_O_4_ in the “OFF” state had a stronger effect than HAw in the “ON” state on cell proliferation and differentiation. We assumed that the mechanical stimulation from a local magnetic field probably actuated via the Fe_3_O_4_ nanoparticles on different HAw/Fe_3_O_4_ fiber rods even in the absence of a magnetic field. Furthermore, the synergistic effect via incorporation of the magnetic HAw/Fe_3_O_4_ and MF stimulation significantly improved gene synthesis during the initial stage of osteanagenesis and induced BMSCs differentiated to produce osteogenesis‐related extracellular matrix, suggesting that the synergistic application of MF and the magnetic HAw/Fe_3_O_4_ composite extended far beyond the effects achievable by either individual component alone. In the presence of MF, the magnetic Fe_3_O_4_ could convert the external MF stimuli into a local mechanical stimulation and simultaneously amplified stimuli signal, that in turn enhanced osteogenesis. It was clear that the integration of HAw/Fe_3_O_4_ and an MF amplified the mechanical stimuli and further synergistically accelerated bone repair reflected in the cell biocompatibility and osteoblastic differentiation, suggesting that the combined utilization of external (MF) and internal (HAw/Fe_3_O_4_) magnetism was a practical method to promote osteogenic efficacy.

### 2.6 Bioinformatics Analysis and Mechanotransduction Mechanism in Magnetic HAw/Fe_3_O_4_

**Figure 8:**
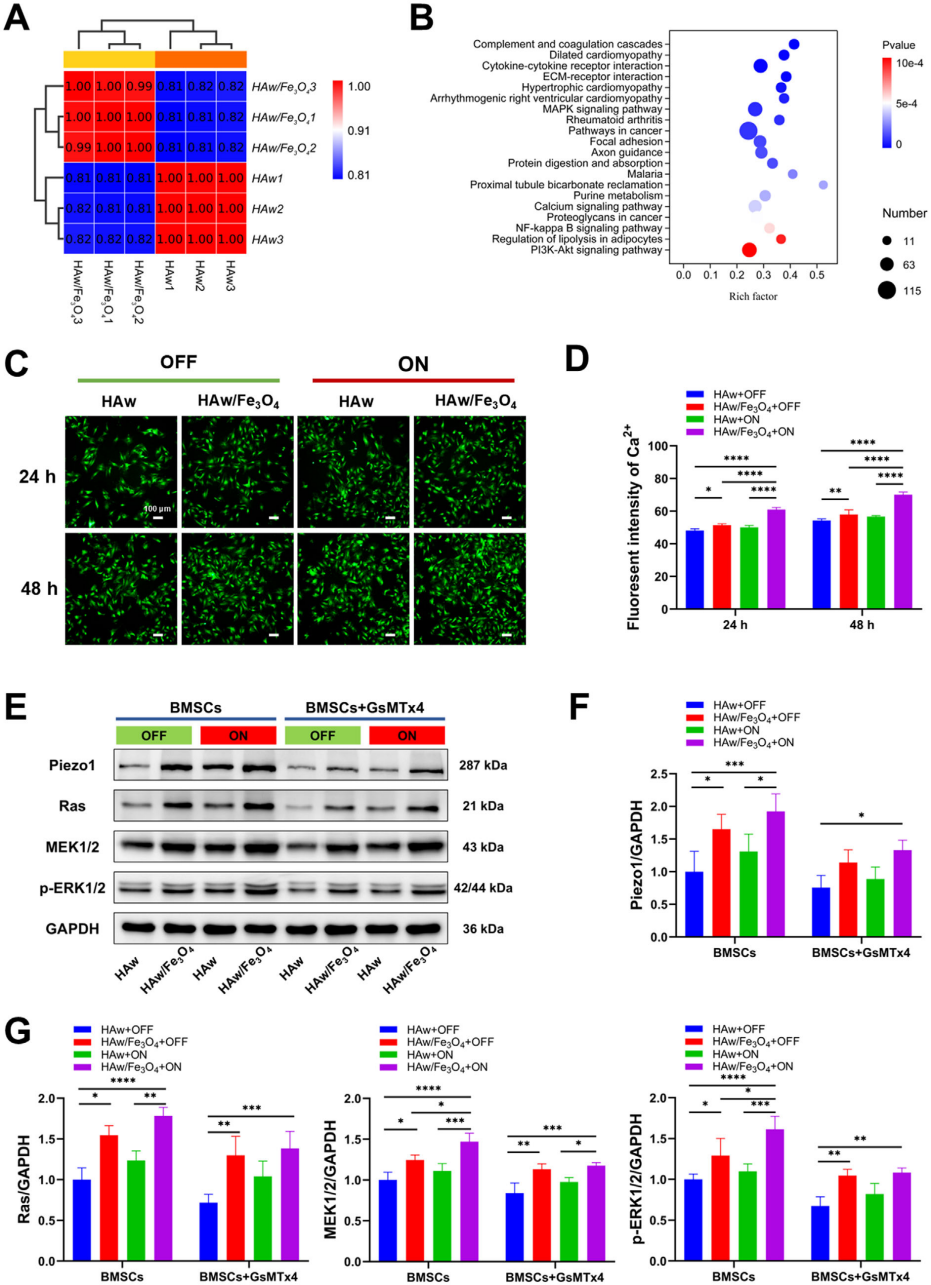
Bioinformatic and mechanistic analysis of BMSCs differentiation with HAw and the magnetic HAw/Fe_3_O_4_ under the activation of an MF. A) Heatmap of Pearson correlation between BMSCs co‐cultured with HAw and the magnetic HAw/Fe_3_O_4_ in the MF. B) Enriched KEGG pathways of HAw versus the magnetic HAw/Fe_3_O_4_ under MF stimulation. C) Fluorescence and D) the fluorescent intensity values of the intracellular Ca^2+^ with BMSCs cultured on HAw and the magnetic HAw/Fe_3_O_4_ via different MF patterns (“OFF” and “ON”) for 24 and 48 h (*n* = 4). E) Western blot analysis and F) the corresponding quantification of Piezo1 and G) MAPK pathway‐related proteins with/without inhibitor (GsMTx4) of Piezo1 via different MF patterns for 7 days (*n* = 3). ^*^ *p* < 0.05; ^**^ *p* < 0.01, ^***^ *p* < 0.001, and ^****^ *p* < 0.0001.

A series of consequences displayed that the synergistic effect of integrating Fe_3_O_4_ nanoparticles and MF enhanced the osteoinductivity of pure HAw. However, the specific mechanism of promoting cell differentiation into osteoblasts via the magnetic HAw/Fe_3_O_4_ with MF was still unclear. Transcriptome sequencing was conducted to test the potential mechanism of cell osteogenic differentiation induced by the magnetic HAw/Fe_3_O_4_ under MF. The Pearson correlation between two groups (magnetic HAw/Fe_3_O_4_ vs. HAw) under MF stimulation demonstrated a qualified specimen stability (**Figure** **8**A). KEGG‐based pathway analysis (Figure 7B) suggested expressed genes were enriched into ECM‐receptor interaction, focal adhesion, and mechanosensitive ion channel mechanotransduction related to response the stimulus in nanostructured biomaterials and might have high correlation with magnetically manipulated stimulation of the magnetic HAw/Fe_3_O_4_ on BMSCs. Most encouragingly, the MAPK signaling pathway was simultaneously identified in enriched KEGG pathways, which was indicated to be closely involved in osteoinductivity of BMSCs. As we focused on the difference between the magnetic HAw/Fe_3_O_4_ and HAw in the “ON” state, expressed genes were analyzed the effect of the magnetic HAw/Fe_3_O_4_ on cells. The results demonstrated significant associations between mechanotransduction processes and three key biological components: (i) cell adhesion molecules, (ii) the cytoskeletal system mediating cell‐ECM interactions, and (iii) MAPK signaling pathway activation (Figure 8B). The incorporation of magnetic iron oxide nanoparticles could be employed as stimuli sign to affect the conformational change of mechanical‐sensitive proteins, controlled magneto‐mechanical stimulation on macrophages, and activate cell signaling molecules or ion channels to promote the osteointegration of biomaterials.^[^ ^30^, ^46^, ^47^ ^]^ Transcriptional profiling of differentially expressed genes revealed significant upregulation of Piezo1 in the magnetic HAw/Fe_3_O_4_ group, demonstrating its functional involvement in mechanotransduction process and osteoinductive property of this magnetic material. Furthermore, the enhancement of osteoinductive effect by HAw/Fe_3_O_4_ was probably related to mechanical stimulation in situ, and the MF could exert a twisting force on HAw/Fe_3_O_4_ attaching to the cell membrane. This process adjusted the configuration of receptor complexes and resulted in the cytoskeletal deformation.^[^ ^23^ ^]^ The excellent adhesion of magnetic HAw/Fe_3_O_4_ was the initiation process, activating transmembrane receptors, conducting mechanical transduction, opening mechanosensitive ion channels on the cell membrane, and then triggering the local Ca^2+^ influx. The mechanical transduction process of mechanical stimuli modulated various physiological processes and finally regulates bone homeostasis.

Intracellular calcium (Ca^2+^) signaling, which acts as a secondary messenger toward the activation of osteoinductivity of BMSCs in mechanotransduction. Previous study showed that the intracellular calcium concentration in MC3T3‐E1 cells exhibited a transient increasing trend due to LIPUS stimulation, and Piezo1 could open for intracellular calcium as a mechanosensitive cation (Ca^2+^) channel.^[^ ^34^ ^]^ As Ca^2+^ signaling played a vital role in mechanotransduction and triggering the Ca^2+^‐mediated signaling pathways for the regulation of bone defect repair, and osteogenic differentiation,^[^ ^48^ ^]^ it was essential to explore how magnetic HAw/Fe_3_O_4_ affects Ca^2+^ flux in BMSCs. Fluorescence imaging analysis of the intracellular Ca^2+^ levels was conducted to evaluate the effects of pure HAw and HAw/Fe_3_O_4_ groups on cells. With MF stimulation, the intracellular calcium ion levels of Fluo‐4 AM‐labeled cells on the magnetic HAw/Fe_3_O_4_ group increased (Figure 8C). Meanwhile, the fluorescence intensities of all the groups were quantified, and the result showed higher fluorescent intensity values of the intracellular Ca^2+^ on the magnetic HAw/Fe_3_O_4_ groups compared with pure HAw groups (Figure 8D). In addition, the fluorescence intensity confirmed that significant difference between the magnetic HAw/Fe_3_O_4_ groups with MF and the parallel control group without MF. However, there was no obvious difference was observed between HAw with and without MF administration groups. Notably, the magnetic HAw/Fe_3_O_4_ group under MF stimulation had the highest intracellular calcium ion levels of Fluo‐4 AM‐labeled cells. Loading Fe_3_O_4_ nanoparticles played a more important role in causing intracellular calcium fluctuations, and the combined promotion of external (MF) and internal (HAw/Fe_3_O_4_) magnetism amplified this volatility.

Piezo1, as the mechanosensor localized at or near the plasma membrane, could be activated by mechanical stimulation of intracellular calcium and played a vital transduction role to convert a variety of biomechanical stimulations through the Ca^2+^ response from the extracellular environment into intracellular biological signals.^[^ ^49^ ^]^ In the process of mechanical transduction, Piezo1 was an important ligament as a mechanosensor, by which mechanical stimulation transmitted into biochemical signals within the cell to stimulate the downstream osteogenic signaling pathway.^[^ ^50^, ^51^ ^]^ Studies have reported that mechanotransduction was a complex cellular signaling process involving activation of multiple signal pathways and expression of transcription factors.^[^ ^26^, ^52^ ^]^ As is widely known as, ERK1/2 in the MAPK pathway is a signaling molecule to be calcium‐mediated for osteoblast proliferation.^[^ ^34^ ^]^ MAPK signaling was an important transmitter of signals from the cell surface to the interior of the nucleus and has been proven that actively participated in the process of extracellular mechanical signal transduction to initiate the bone repair. The expression of ERK1/2 (the key marker of cell proliferation and differentiation) was exactly detected to be significantly upregulated in the magnetic HAw/Fe_3_O_4_ group mentioned in the expressed genes of the transcriptome sequencing. Collectively, our experimental evidence supported a mechanistic framework whereby the magnetic HAw/Fe_3_O_4_ composite under applied MF enhanced cellular osteogenesis through coordinated activation of the MAPK signaling cascade, with Piezo1 functioning as a critical mechanotransduction mediator in this regulatory axis. To validate this hypothesis, we further analyzed the differential gene expression related to interactions about all them and the upstream signaling pathway mediating MAPK signaling. The expression levels of Piezo1, Ras, MEK1/2, and p‐ERK1/2 in BMSCs were observed using Western blot (Figure 8E). The levels of genes (Piezo1, Ras, MEK1/2, and p‐ERK1/2) were up‐regulated in the magnetic HAw/Fe_3_O_4_ groups compared with the HAw groups, indicating the activation of Piezo1 and MAPK signaling by integrating Fe_3_O_4_ nanoparticles (Figure 8F, G). Furthermore, the levels of MEK1/2, and p‐ERK1/2 were also up‐regulated in the magnetic HAw/Fe_3_O_4_ group under MF stimulation compared to parallel group without MF, whereas no difference in HAw whether to use an MF. In this study, protein expression levels of Piezo1, Ras, MEK1/2, and p‐ERK1/2 proteins were significantly increased immediately at the magnetic HAw/Fe_3_O_4_ with the “OFF” state and attained a highest level in the “ON” state, indicating that mechanical stimulation from a local magnetic field actuated via the Fe_3_O_4_ nanoparticles and synergetic intensive stimulation via MF and the magnetic HAw/Fe_3_O_4_ in BMSCs. Next, we conducted a series of rescue experiments via employing GsMTx4^[^ ^53^ ^]^ as an inhibitor of Piezo1 to explore the regulatory relationship at the MAPK pathway (Figure 8E). GsMTx4 was a spider venom peptide, acting in selective inhibition of cationic‐permeable mechanosensitive channels relevant to the Piezo channel families.^[^ ^54^ ^]^ The change of the expression levels (Piezo1, Ras, MEK1/2, and p‐ERK1/2) with application of Piezo1 inhibition was further verify. Compared with parallel control groups without inhibitor application, GsMTx4 could attenuate the MAPK pathway activation stimulated and significantly reduce the protein expression levels of Piezo1, Ras, MEK1/2, and p‐ERK1/2 in all groups (Figure 8F,G). In the study, the expression levels (MEK1/2 and p‐ERK1/2) of the magnetic HAw/Fe_3_O_4_ group were significantly increased by exerting MF stimulation (Figure 8G). However, in the GsMTx4 groups, the increase was not obvious at the magnetic HAw/Fe_3_O_4_ group in the “ON” state, compared to the same parameter in the “OFF” state. The experimental data conclusively demonstrated that Piezo1 mediates mechanotransduction through integration with Fe_3_O_4_ nanoparticles under applied MF, while concurrently regulating MAPK signaling cascades that were potently activated during osteogenic differentiation. Our findings deciphered a magneto‐chemomechanical coupling paradigm wherein: (i) Calcium ions functioned as secondary messengers mediating HAw/Fe_3_O_4_‐driven magnetomechanical stimulation under applied MF to initiate differentiation commitment, and (ii) Piezo1 served as a mechanosensor converting magnetic stimuli into MAPK signaling activation, thereby orchestrating the osteogenic differentiation program in BMSCs.

### 2.7 Osteoinductivity of Magnetic HAw/Fe_3_O_4_ and Immunofluorescence Staining In Vivo

**Figure 9:**
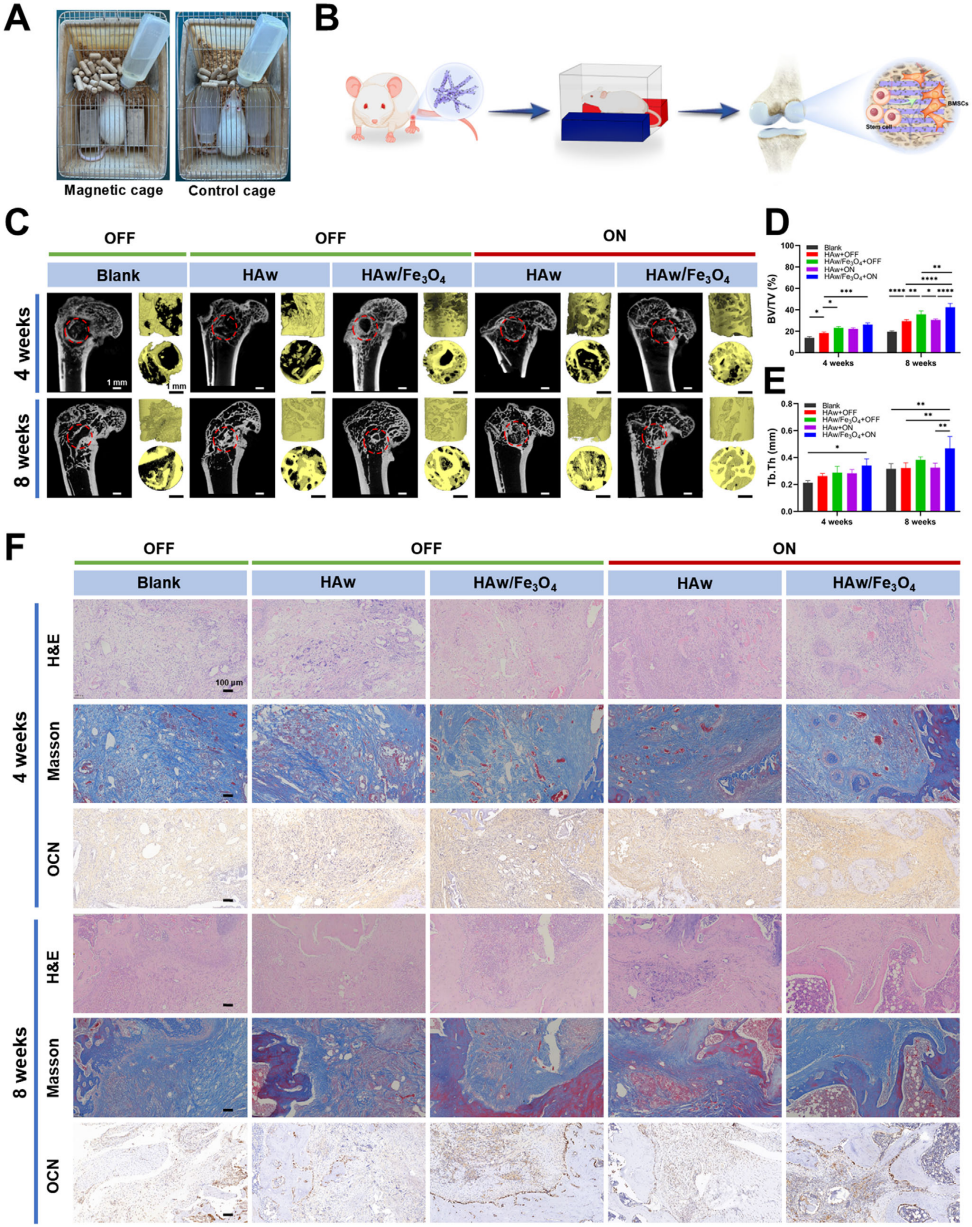
Osteoinductivity of the magnetic HAw/Fe_3_O_4_ under the MF stimulation in vivo. HAw and the magnetic HAw/Fe_3_O_4_ were grafted into the bone defect by different MF patterns (“OFF” and “ON”) for 4 and 8 weeks. A) Photographs of the designed magnetic field device. B) A schematic of the surgery processes for the rat femur model. C) CT images and reconstructed 3D tomography of the repaired rat femur. D) Total volume of bone tissue‐like components (BV/TV) and (E) trabecular thickness (Tb.Th) in the defect region. F) H&E, Masson staining, and immunohistochemistry staining (OCN) of the bone defect area. ^*^ *p* < 0.05; ^**^ *p* < 0.01, ^***^ *p* < 0.001, ^****^ *p* < 0.0001, *n* = 3.

The rat femur defect model was selected to evaluate the osteoinductivity of HAw and magnetic HAw/Fe_3_O_4_ (**Figure** **9**A,B). After 4 and 8 weeks of implantation, new bone formation of the bone defects was assessed by Micro‐CT. For histomorphometric analysis, mid‐sagittal sections of cylindrical defects were processed using standardized protocols and stained with H&E, Masson's trichrome, and immunohistochemistry staining targeting OCN. To further investigate the bone repair property of the magnetic HAw/Fe_3_O_4_, immunofluorescence staining for OCN and Col‐1 was performed. The results identified via 3D reconstruction revealed that low‐density images with minimal bone ingrowth were detected in the blank group, while bone defects implanted with bio‐materials displayed improving repair images compared to the blank group either at 4 or 8 weeks (Figure 9C). In comparison to the pure HAw groups, the bone defect areas showed higher image densities in the magnetic HAw/Fe_3_O_4_ groups. Moreover, bone tissue‐like components in the bone defects of the magnetic HAw/Fe_3_O_4_ in the “ON” state were higher than that in “OFF” state. Quantitative data were calculated including the percent bone volumes (BV/TV) and trabecular thickness (Tb.Th) to evaluate bone regeneration (Figure 9D,E). The results revealed higher BV/TV and Tb. Th in the magnetic HAw/Fe_3_O_4_ groups compared to the pure HAw groups (Figure 9D), and the numerical value of the magnetic HAw/Fe_3_O_4_ in the “ON” state was higher than that in the “OFF” state at 8 weeks. Most impressive bone repair was exhibited in the magnetic HAw/Fe_3_O_4_ groups with MF stimulation, suggesting that the magnetic HAw/Fe_3_O_4_ with an MF promoted osteogenesis. However, there were no significant differences in Tb.Th between HAw in the “OFF” state and the blank, and no significant differences in BV/TV and Tb.Th were noticed between HAw in the “ON” state and in the “OFF” state, indicating the limited effect of osteoinductivity via pure HAw with or without MF stimulation.

H&E and Masson's trichrome staining showed that the defect sites in the blank group were filled by loose fibrous tissues (Figure 9F) at week 4. Initial bone migration and formation of the magnetic HAw/Fe_3_O_4_ was greater than that of pure HAw. New bone formation in the blank group, HAw “OFF” group, and HAw “ON” group was limited, but that was remarkably promoted in the magnetic HAw/Fe_3_O_4_ in the “ON” state possessing minimal bony islands at the defect margin. Increased new bone formation in the magnetic HAw/Fe_3_O_4_ groups compared to the pure HAw groups, and the magnetic HAw/Fe_3_O_4_ in the “ON” state generated a significant bone repair response at week 8. Moreover, the magnetic HAw/Fe_3_O_4_ composite with applied MF (“ON” state) demonstrated progressive neo‐bone formation extending radially from the defect periphery, correlating with significantly enhanced OCN expression levels through immunohistochemical analysis. The magnetic HAw/Fe_3_O_4_ composite under external MF demonstrated the largest volume of OCN‐positive tissue formation, indicating a synergistic enhancement of bone regeneration through coordinated mechanobiological regulation. Histological analysis in vivo indicated that the highest levels of defect coverage and bone volume were observed in the magnetic HAw/Fe_3_O_4_ group under MF stimulation. Consistent with the results of micro‐CT, histological staining confirmed that the magnetic HAw/Fe_3_O_4_ group under MF stimulation generated a significant bone repair response. In the control blank group, excessive fiber encapsulation and delayed union were observed, while inordinately growing bony repair was detected in the materials implanted groups. Compared to the control blank group and HAw groups, the group of magnetic HAw/Fe_3_O_4_ in the “ON” state showed the largest area of the newly formed bone, indicating synergistic promoting effects of surface magnetic modification and MF on osteogenesis. Moreover, the newly formed bone was thicker and more mature located at the bone defect margin, and growing bony islands dispersed in the fibrous tissue within bone fractures boosting bone ingrowth in the magnetic HAw/Fe_3_O_4_ with an MF, thus leading to a better bone regenerative effect.

**Figure 10:**
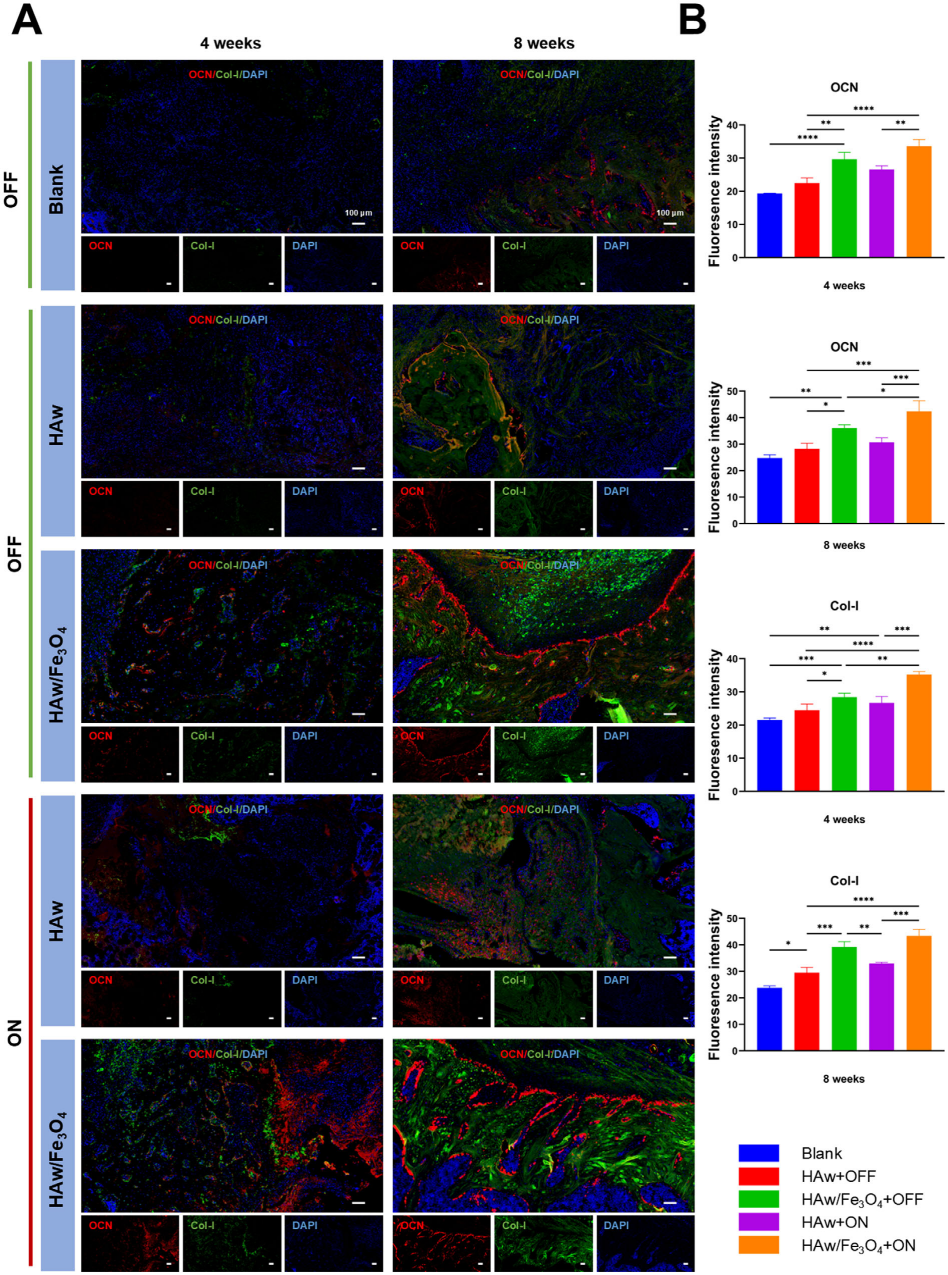
Histological evaluations of osteogenesis. A) Representative immunofluorescence images of OCN, Col‐I, and cell nuclei stained by different MF patterns (“OFF” and “ON”) in vivo at 4 and 8 weeks. B) Quantitative analysis of all positively stained areas. ^*^ *p* < 0.05; ^**^ *p* < 0.01, ^***^ *p* < 0.001, and ^****^ *p* < 0.0001, *n* = 3.

Importantly, immunofluorescence staining for OCN and Col‐I further supported these findings (**Figure** **10**A). The magnetic HAw/Fe_3_O_4_ in the “ON” state exhibited larger bulk of newly formed bone and a larger region of OCN‐positive and Col‐I‐positive tissue than parallel control in the “OFF” state and HAw groups, that was consistent with the results in vitro. Similar results were also verified in quantitative measurement of immunofluorescence staining for OCN and Col‐I (Figure 10B). There were significant differences between HAw and the magnetic HAw/Fe_3_O_4_ in the “ON” state, and significant differences were also noticed between the magnetic HAw/Fe_3_O_4_ at the “OFF and ON” state in OCN at 8 weeks and Col‐I at 4 weeks. However, no significant differences were found between HAw in the “OFF” state and in the “ON” state. This discrepancy revealed that MF significantly promoted osteogenic induction of the magnetic HAw/Fe_3_O_4_, whereas the effect was unremarkable to HAw in vivo. The results identified magnetic modification increased the osteoinductivity of HAw in vivo, and the magnetic HAw/Fe_3_O_4_ could promote new bone formation, especially under MF stimulation. Moreover, no obvious pathological changes in brain, lung, heart, liver, spleen, and kidney tissues were perceived, indicating good biocompatibility of the magnetic HAw/Fe_3_O_4_ in vivo (Figure S6, Supporting Information).

The previous finding certified that 3D‐printed porous Ti scaffolds through a magnetic Fe_3_O_4_/polydopamine (PDA) coating under an external SMF improved bone regeneration in vivo.^[^ ^55^ ^]^ It was already clear that integration of the scaffold and the SMF synergistically expedited osteogenesis. In this study, all these data analogously implied that magnetic Fe_3_O_4_ nanoparticles modified HAw could accelerate bone repair and increase bone strength by promoting the osteogenesis of femur defect. Meanwhile, the results tentatively figured out the mechanical signal transduction mechanisms of Piezo1 channel activation via nano‐Fe_3_O_4_ particles modified HAw for optimizing osteogenic capacity of stem cells. Taken together, the following factors are a few considerations that probably contributed to the enhancement of osteogenic capacity by the magnetic HAw/Fe_3_O_4_. First, surface modification with the Fe_3_O_4_ nanoparticles on HAw/Fe_3_O_4_ provided the adhesion anchors and increased the adhesion area, that was conducive to cell adhesion and proliferation. Second, the special structure (micron‐sized HAw and nano‐sized Fe_3_O_4_ particles) provided good interaction for cells and further improve osteogenic differentiation. Third, the combined application of a local magnetic field actuated via the Fe_3_O_4_ nanoparticles and an external magnetic field expanded the mechanical stimulation for cells to precisely regulate in situ bone regeneration.

Notwithstanding the promising results, several limitations exist in the current investigation. Our study primarily investigated using magnetic HAw/Fe_3_O_4_ to deliver mechanical stimuli via an MF, aiming to enhance the osteogenic differentiation of BMSCs. However, the potential for spatial and temporal variability in the magnetic field strength experienced by cells during exposure could arise from factors such as minor power source fluctuations, sample positioning relative to the field source, or inherent field gradients. To ensure highly precise and reproducible stimulation, we will implement a well‐calibrated static field system to strengthen the reliability and interpretability of cellular responses to MFs in our upcoming experimental studies. Moreover, the combined application of HAw/Fe_3_O_4_ and an MF amplified the mechanical stimuli and further synergistically enhanced osteogenic efficacy. The mechanism underlying the Fe_3_O_4_ itself and MF‐induced osteogenic enhancement requires clarification in subsequent studies. Our results indicated that Piezo1 served as a mechanosensor and MAPK/ERK signaling cascades mediating HAw/Fe_3_O_4_‐driven magnetomechanical stimulation under an MF to initiate differentiation commitment. RhoA/ROCK and YAP/TAZ are important transmitters participated in the process of mechanical stimulus transduction. Inhibiting RhoA/ROCK or YAP/TAZ will be useful for exploring the dominant signaling mechanism. Furthermore, we still need to further investigate the mechanical forces delivered at the correct subcellular location and further explore the membrane localization of the nanoparticle and the claim of Piezo1‐mediated effects.

## 3 Conclusion

In summary, this study pioneered a magneto‐active bone regeneration platform through the strategic integration of Fe_3_O_4_ nanoparticles with HAw. The engineered HAw/Fe_3_O_4_ composite preserved the intrinsic physicochemical profile of native HAw while acquiring dynamic magnetic responsivity, enabling: (i) programmable microstructural alignment along applied MF vectors, and (ii) amplified mechanobiological cue delivery for precise cellular modulation. Biocompatibility assays confirmed Fe_3_O_4_ nanoparticles integration enhanced bioactivity compared to unmodified HAw. Through synchronized MF stimulation, the composite demonstrated significant osteoinductive potential and promoted bone tissue regeneration in vitro and in vivo. Mechanistically, we established Piezo1‐mediated mechanotransduction as the central axis orchestrating downstream MAPK/ERK signaling cascades in response to magneto‐mechanical inputs. This work provided a paradigm‐shifting approach for spatiotemporally controlled bone reconstruction through rational design of smart biomaterial‐magnetic interfaces.

## 4 Experimental Section

### Synthesis of HAw

HAw was prepared by the modified hydrothermal homogeneous precipitation method according to previous reports.^[^ ^11^ ^]^ A mixture of Ca (NO_3_)_2_•4H_2_O and (NH_4_)_2_HPO_4_, using CON_2_H_4_ as a precipitant and in the presence of C_6_H_14_O_6_ as a template, was dissolved in 400 ml of deionized water with continuous mechanical stirring for 10 min. After the solutions were mixed, the concentration of Ca was 0.15 mol L^−1^ and the atomic ratio of Ca/P was 1.67. Meanwhile, the addition amount of CON_2_H_4_ was 24 g, and C_6_H_14_O_6_ accounted for 1% of Ca (NO_3_)_2_•4H_2_O. Then, HNO_3_ was added dropwise into the reaction mixture in a small‐mouth sealed bottle until the pH was reduced to 2.5 and kept in a 95 °C incubator for 24 h. The synthesized HAw was washed until neutral. The filtrated final products were obtained by lyophilization.

### Preparation of Magnetic HAw/Fe_3_O_4_

4% sodium oleate was dissolved in ultra‐pure water at 85 °C, and then 4 g HAw was added to the above solution with a continuous stirring for 2 h at 85 °C. After cooling to room temperature, the sample was immersed in distilled water to remove excess sodium oleate and dried at 60 °C. The composite and iron acetylacetone (Fe(acac) 3) were added into a beaker containing 90 ml triethylene glycol. And then the mixed solution was stirred and ultrasonically dispersed for 60 min. The dispersed suspension treated with nitrogen in 150 ml stainless steel hydrothermal reactor lined with polytetrafluoroethylene for 15–20 min and kept at 220 °C for 12 h. The products were naturally cooled to room temperature, repeatedly washed with anhydrous ethanol. Sodium oleate was added into the alcohol and the solution was heated to 58 °C with a continuous stirring. Then, the obtained products were added into the solution and stirred for 2 h. Finally, after natural cooling, the filtrated solid products were obtained by drying. The synthesized HAw and HAw/Fe_3_O_4_ were sterilized under ultraviolet light for 30 min before cell culture.

### Characterization

The surface morphology of HAw/Fe_3_O_4_ and HAw, the surface element distribution of HAw/Fe_3_O_4_, and the arrangement of HAw/Fe_3_O_4_ under an MF were analyzed by SEM‐EDS (SEM, Zeiss). The crystalline structures of pure HAw and HAw/Fe_3_O_4_ were recognized using X‐ray diffraction (XRD, Rigaku), and the Jade9 software was used for quantitative analysis of the content of each phase, crystallinity of crystal planes, and grain size in HAw/Fe_3_O_4_. To further confirm the presence of ferrous ferroferric oxide (Fe_3_O_4_) in HAw/Fe_3_O_4_ and the maintenance of the main structure of HAw, Fourier transform infrared spectroscopy (FTIR, Thermo Scientific) was used to analyze the functional groups contained in the material. Furthermore, the magnetization hysteresis (*M−H*) curve of HAw/Fe_3_O_4_ was detected by superconducting quantum interference devices (Quantum Design MPMS 3). A vibrating sample magnetometer (VSM; Lake Shore 7404) was used to measure the hysteresis curves of HAw/Fe_3_O_4_. A magnet was placed near the HAw/Fe_3_O_4_ solution to verify the magnetic properties, and the overall arrangement of HAw/Fe_3_O_4_ and HAw with and without an external MF was observed by optical microscopy.

### Application Condition Assay—Isolation and Culture of BMSCs

Bone marrow mesenchymal stem cells (BMSCs) were isolated according to a previously described protocol with proper modification.^[^ ^45^ ^]^ Briefly, 4‐week‐old male Sprague‐Dawley (SD) rats were euthanized, and the bone marrow was flushed from the marrow cavity of the femurs and tibiae with collected in Dulbecco's modified Eagle's medium (Hyclone, USA) supplemented with 10% fetal bovine serum (FBS, Gibco, USA) under sterile conditions. The bone marrow, which was centrifuged at 1000 rpm for 5 min after lysis of red blood cells, was resuspended and cultured in DMEM supplemented with 10% FBS and 1% penicillin/streptomycin (Gibco, USA). Cells were incubated under a humidified incubator at 5% CO_2_ and 37 °C, and nonadherent cells were removed. After 7 days of culture, more than 95% pure BMSCs were obtained. The complete medium was typically replaced every 2 days, and BMSCs were sub‐cultured via trypsinization at 70–80% confluence. The third‐generation BMSCs were obtained for subsequent co‐culture experiments. The medium was in the presence of ascorbic acid, β‐glycerophosphate, and dexamethasone (Sigma–Aldrich, USA) for osteoblast differentiation.

### Application Condition Assay—Concentrations Assay

A cell counting assay kit‐8 (CCK‐8) assay was used to evaluate in vitro cytocompatibility of HAw/Fe_3_O_4._ For chosen of the optimal co‐cultured concentrations of HAw/Fe_3_O_4_, BMSCs were seeded into HAw/Fe_3_O_4_ with gradient concentrations (0, 5, 10, and 20 µg mL^−1^ HAw/Fe_3_O_4_) for application. BMSCs were placed into 96‐well plates at a density of 5 × 10^3^ per well for 24 h and then cultured in 0, 5, 10, and 20 µg mL^−1^ HAw/Fe_3_O_4_, respectively. Cells cultured without material supplementation (0 µg mL^−1^) functioned as the control cohort. After cultured for 24, 48, and 72 h, the supernatants were abandoned and fresh medium containing 10% CCK‐8 reagent (Beyotime, China) was added to each well. After 4 h incubation 37 °C, optical density (OD) at 450nm was detected by a spectrophotometer (Tecan, Austria). Cell viability was calculated according to the following formula:

(1) $$\begin{array}{ccc} \mathrm{Cell}\;\mathrm{viability}\left(\%\right) & = & \left(\mathrm{OD}\;\mathrm{experimental}\;\mathrm{group}-\mathrm{OD}\;\mathrm{blank}\;\mathrm{group}\right)/\\ & & \;\left(\mathrm{OD}\;\mathrm{control}\;\mathrm{group}-\mathrm{OD}\;\mathrm{blank}\;\mathrm{group}\right)\times 100\% \end{array}$$

Alkaline Phosphatase (ALP) quantitative assay and ALP staining were performed to detect the osteogenic differentiation potential about gradient concentrations of HAw/Fe_3_O_4_, and then determined the optimal application concentrations. BMSCs were placed into 24‐well plates at a density of 5 × 10^4^ per well. Cells were co‐cultured with 0, 5, 10, and 20 µg mL^−1^ HAw/Fe_3_O_4_ in differentiation medium for 7 and 14 days. At the corresponding time, the cells were rinsed with PBS three times, and lysed with CelLytic Buffer (Sigma‐Aldrich, USA). After centrifuged, cells of each species were incubated with an ALP kit (Beyotime, China) at 37 °C. The optical density was measured by a microplate reader (Tecan, Austria) at 405 nm. The quantitative assay data was normalized to a total protein basis of the manufacturer's guidelines using an Enhanced BCA Protein Assay Kit (Beyotime, China). For ALP staining, cells were co‐cultured with gradient concentrations of HAw/Fe_3_O_4_ in conditioned medium. After cultured for 7 and 14 days, cells of each species were fixed with 4% paraformaldehyde for 30 min, and then stained with a BCIP/NBT ALP Color Development Kit (Beyotime, China).

### Application Condition Assay—MF Stimulation

The MF stimulation system in vitro included an acrylic frame and two rectangle nickel‐plated neodymium (Nd2Fe14B) permanent magnets, as shown in Figure 4A,B. For evaluating the optimal MF stimulation intensity, BMSCs were incubated in the device with different stimulation intensities (Figure 4C) using ALP assay. The permanent magnets (15, 20, 25 mm thick) with different intensities (150, 270, and 350 mT) were embedded in the inter‐layer on both sides of the device for groups T1, T2, and T3, respectively. Group T0 served as the non‐magnetic control. For chosen of the optimal MF stimulation mode (intermittent or continuous stimulation), BMSCs were incubated in the device with different stimulus durations (4, 24h day^−1^, Figure 5D) labelled as “ON/OFF” and “ON” using CCK‐8 assay and ALP assay. The same apparatus without two magnets was as the control group marked with the “OFF” groups. All assays were performed to determine the optimal MF stimulation application for the follow‐up tests.

### In Vitro Cell Assay—Cell Morphology on Magnetic HAw/Fe_3_O_4_

Aim to appraise the adaptation of BMSCs to the magnetic HAw/Fe_3_O_4_ in MF stimulation, the cells were stained for the actin filaments and nucleus after 24 and 48 h of culture. The co‐cultured concentrations and MF stimulation for cell experiments of materials in vitro were determined by the assays as described above. Briefly, BMSCs were seeded in 24‐well plates at a density of 1 × 10^5^ per well for 24 h and then co‐cultured with the samples (10 µg mL^−1^) of HAw and HAw/Fe_3_O_4_. The previous designed MF device of 270 mT was implemented to the HAw and HAw/Fe_3_O_4_ to act as the MF stimulation (4h day^−1^) on BMSCs marked with the “ON” groups (Figure 6A). Cells cultured on HAw and HAw/Fe_3_O_4_ without MF stimulation served as the control marking with the “OFF” groups. After washing the samples with PBS 2 times, the cells were fixed using a 4% (v/v) formaldehyde solution (Sigma‐Aldrich, USA) for 10 min and then permeabilized with PBS solution containing 0.5 % TritonX‐100 at room temperature for 5 min. Subsequently, the samples were staining using fluorescein‐labeled phalloidin solution (Beyotime, China).

### In Vitro Cell Assay—Cell Proliferation and Viability Assay

CCK‐8 assay was used to evaluate cell viability in vitro_._ BMSCs were digested and seeded in 96‐well plates at a density of 5 × 10^3^ per well. The materials (10 µg mL^−1^) of HAw and HAw/Fe_3_O_4_ were added to cells, respectively. Cells cultured on HAw and HAw/Fe_3_O_4_ with or without MF stimulation (270 mT, 4h day ^−1^) for 24, 48, and 72 h, and then the optical density (OD) at 450nm was detected by a spectrophotometer (Tecan, Austria).

### In Vitro Cell Assay—ALP Assay and Matrix Mineralization

Groups were the same as described above in the “CCK‐8” sections. For ALP assay, BMSCs were seeded at 5×10^4^ cells well^−1^ in 24‐well plates. Cells were cultured on HAw and HAw/Fe_3_O_4_ with or without MF stimulation for 3 and 7days. At the point of detection, the optical density of all samples was measured by a microplate reader (Tecan, Austria) at 405 nm, and the cells for ALP staining were stained with a BCIP/NBT ALP Color Development Kit (Beyotime, China).

Alizarin red S (ARS) staining was performed to inspect matrix mineralization and calcium deposition. The cells were seeded at 5×10^5^ cells well^−1^ in 6‐well plates and then co‐cultured with 10 µg mL^−1^ HAw/Fe_3_O_4_ and HAw in differentiation medium with or without MF stimulation. After induction for 14 and 28 days, the BMSCs were fixed and stained with ARS staining kit (Beyotime, China) for 30 min. Following the staining desorbed using 10% cetylpyridinium chloride destain solution, the absorbance at 562 nm was measured.

### In Vitro Cell Assay—Fluorescence of the Intracellular Calcium Ion Levels Detection

Groups were the same as described above in the “CCK‐8” sections. The calcium ion concentration in BMSCs was evaluated by dye Fluo‐4 AM (Beyotime, China). Briefly, the cells were seeded at 5×10^4^ cells well^−1^ in 24‐well plates, and co‐cultured on 10 µg mL^−1^ HAw/Fe_3_O_4_ and HAw with or without MF stimulation for 24 and 48 h. Subsequently, the cells were washed 2 times with PBS and then stained with Fluo‐4 AM solution in darkness for 50 min. Finally, fluorescence imaging of the intracellular calcium ions was detected using a fluorescence microscope. The fluorescent intensity values of the intracellular Ca^2+^ were calculated by Image J software.

### In Vitro Cell Assay—Real‐Time PCR

The four groups with or without MF were divided as described above. After BMSCs (4×10^5^ cells) were cultured with differentiation medium in 6‐well plates for 3 and 7 days, total cellular RNA of each sample was extracted from cells using TRIzol reagent (Takara, Japan). The cDNA was prepared using the HiScript II Q RT SuperMix for qPCR (Vazyme, China) in the light of the manufacturer's protocols. The expression levels of ALP, runt‐related transcription factor 2 (Runx2), type I collagen (COL‐I), and osteocalcin (OCN) were conducted using a 7500 real‐time PCR system (Applied Biosystems, USA) with ChamQ Universal SYBR qPCR Master Mix (Vazyme, China). Glyceraldehyde‐3‐phosphate dehydrogenase (GAPDH) was used as the reference gene. The primer sequences were shown in Table S4 (Supporting Information). The relative fold change of gene expression was calculated by the 2^‐∆∆Ct^ method.

### In Vitro Cell Assay—RNA Sequencing and Bioinformatics Analysis

For RNA sequencing, total RNA was extracted from BMSCs co‐cultured on the HAw and HAw/Fe_3_O_4_ under MF after 7 days using TRIzol reagent (Takara, Japan). The cell lysates were stored at −80 °C for later analysis. The products were sequenced using an Illumina NovaSeq 6000 platform (Illumina, USA), and a comparative sequencing analysis was performed between the expression levels of miRNAs and the bioinformatics miRNA database according to previously methods.^[^ ^35^ ^]^ All the data were implemented in functional enrichment analysis using Kyoto Encyclopedia of Genes and Genomes (KEGG) pathway analysis.

### In Vitro Cell Assay—Western Blot Analysis

For verifying cellular mechanisms and the role of Piezo1, BMSCs were seeded at a density of 4 × 10^5^ per well in 6‐well plates, and co‐cultured on HAw and HAw/Fe_3_O_4_ with or without MF stimulation. After the cells were collected at 7 days, total protein was extracted by RIPA, separated by 10% SDS‐PAGE, and transferred onto PVDF membranes (Millipore, USA) at 80 V for 90 min. The membranes were blocked with 5% BSA for 1h and then incubated with primary antibodies against Piezo1 (Affinity, Australia), Ras (CST, USA), MEK1/2 (Boster, China), p‐ERK1/2 (CST, USA), and GAPDH (Affinity, USA) overnight at 4 °C. Simultaneously, inhibitor (GsMTx4 TFA, TargetMol, USA) of Piezo1 was used for the explored of Piezo1 in BMSCs. The MAPK signaling pathway mediated by Piezo1 was further detection by western blot at 7days. Then, the membranes were incubated with horseradish‐peroxidase (HRP)‐labeled secondary antibodies (Boster, China) for 2h, and the targeted proteins were detected using an enhanced chemiluminescence detection method. The data from all groups were normalized with the GAPDH protein. The relative expression of the target protein was performed by Image J software.

### In Vivo Biological—Animal Model Establishment, and Materials Implantation

All animal experiments were approved by the Medical Ethics Committee of China Medical University and Stomatology Hospital (K2023055). The average weight of 8‐week‐old male Sprague–Dawley rats (Changsheng Biotechnology, China) was 250 g. The rats were randomly divided into five groups (n = 6) mentioned above for implantation. A cylindrical non‐penetrating bone defect with a diameter of 3 mm and a depth of 3 mm was prepared on the lateral condyle of the rat femur to assess osteogenic effects. HAw and HAw/Fe_3_O_4_ were randomly implanted into the bone defect in powder form. The MF stimulation system in vivo as previously described consisted of an acrylic frame and two cuboid permanent magnets (100 × 50 × 45 mm), as shown in Figure 9A, B. The strength of the MF in the device was ≈270 mT, similarity to the design in vitro. The space of the frame was fabricated to make sure the rats were unable to turn around or escape from the device. The cuboid polyethylene bottles replaced the permanent magnets as a no‐magnet control device. All rats were raised in well design MF devices or no‐magnet apparatuses. The rats were fed in cages without MF stimulation during the whole test, marked with the “OFF” group. Rats of the “ON” group were implemented with MF for 4 h every day. Femurs drilled only ‐ without material implantation or MF stimulation ‐ served as the blank group. All animals received a subcutaneous postsurgical injection of penicillin for 3 days and were sacrificed at 4 and 8 weeks after implantation.

### In Vivo Biological Properties—Micro‐CT Analysis

All the rat femur specimens were collected, fixed using paraformaldehyde, and scanned by Micro‐CT (Scanco Medical, Switzerland). The parameter of X‐ray energy was 70 kv / 200 µA, and at a resolution of 14.8 µm to evaluate osteogenesis. The scanning data of samples was analyzed and the 3D reconstruction images was obtained using an image analysis software. To match the density of bone tissue, elements within the VOI were predefined (threshold was 65‐150). According to the dimension of the bone defect, the cylindrical volume of interest (VOI) was set at a diameter of 3 mm and a height of 3 mm in the bone defects. The defect coverage percent was quantified to appraise new bone formation.

### In Vivo Biological Properties—Histology and Immunohistochemistry Staining

The femur samples were fixed with paraformaldehyde and decalcified in 10% EDTA for 4 weeks after micro‐CT scanning. Specimens were dehydrated with graded ethanol solution, embedded in paraffin, and then sectioned into 4‐µm slices. The sections were stained with hematoxylin and eosin (H&E) and Masson's trichrome staining solution (Solarbio Life Sciences, China) for histology. Major viscera, including the brain, lung, heart, liver, spleen, and kidney, were simultaneously stained with H&E staining for evaluating the in vivo biocompatibility of HAw/Fe_3_O_4_. For immunohistochemistry staining, sections of the femur samples were treated with primary antibodies against OCN (1:200, Servicebio, China) followed by corresponding secondary antibodies conjugated to HRP. All the sections were scanned by a digital scanner (Shengqiang, China).

### In Vivo Biological Properties—Immunofluorescence Staining

After rinsed with PBS and blocked with goat serum, the sections were incubated overnight at 4 °C using primary antibodies against type‐I rabbit collagen (Col‐I) and osteocalcin (OCN). Subsequently, the slices were incubated with the appropriate secondary antibodies for 60 min (1:100, Beyotime, China) in the dark, and stained with DAPI for 5 min. Fluorescent signals were scanned by a digital scanner, and the acquired images were then subjected to quantitative analysis using ImageJ software.

### Statistical Analysis

Numerical data were expressed as mean values ± standard deviation (mean ± SD). Results were analyzed by student's *t‐test* for two groups comparisons. The statistical differences of multiple groups were assessed by one‐way analysis of variance (ANOVA). The significant differences were denoted using asterisks (^*^ *p* < 0.05; ^**^ *p* < 0.01, ^***^ *p* < 0.001, ^****^ *p* < 0.0001, and ns, no significance).

## Conflict of Interest

The authors declare no conflict of interest.

## Supporting information



Supporting Information

## Data Availability

The data that support the findings of this study are available from the corresponding author upon reasonable request.
