# Supplementary material for: Magnetic Fe3O4 Nanoparticles Modified Hydroxyapatite Whisker: A Novel Framework with Superior Osteogenic Efficacy
Source: Adv Sci (Weinh). 2025 Aug 11;12(39):e09715. doi: 10.1002/advs.202509715 (PMC12533391; doi:10.1002/advs.202509715)
Supplement: Supplementary file 1 — Supporting Information [file ADVS-12-e09715-s001.docx]

**Supporting Information**

**Table S1.** FWHM and grain size of the main crystal planes of hydroxyapatite before and after Fe_3_O_4_ loading.

| **Crystal face/2θ** | **FWHM/HAw** | **D/nm** | **FWHM/HAw/Fe_3_O_4_** | **D/nm** |
| --- | --- | --- | --- | --- |
| 100 (10.820) | 0.363 | 22.9 | 0.144 | 76.8 |
| 211 (31.773) | 0.381 | 22.5 | 0.146 | 77.3 |
| 112 (32.196) | 0.264 | 33.8 | 0.194 | 49.4 |
| 300 (32.902) | 0.356 | 24.2 | 0.189 | 51.8 |
| 310 (39.818) | 0.342 | 25.8 | 0.200 | 48.6 |
| 222 (46.711) | 0.353 | 25.6 | 0.199 | 50.3 |
| 410 (51.283) | 0.380 | 24.0 | 0.160 | 70.2 |

**Table S2.** Crystallinity of the main crystal planes of hydroxyapatite before and after Fe_3_O_4_ loading.

| Crystal face/2θ | Crystallinity/HAw (%) | Crystallinity/HAw/Fe_3_O_4_ (%) |
| --- | --- | --- |
| 100 (10.820) | 96.50 | 98.58 |
| 211 (31.773) | 70.80 | 70.37 |
| 112 (32.196) | 97.85 | 96.42 |
| 300 (32.902) | 82.72 | 71.76 |
| 310 (39.818) | 95.29 | 95.88 |
| 222 (46.711) | 96.29 | 95.07 |
| 410 (51.283) | 97.03 | 97.6 |


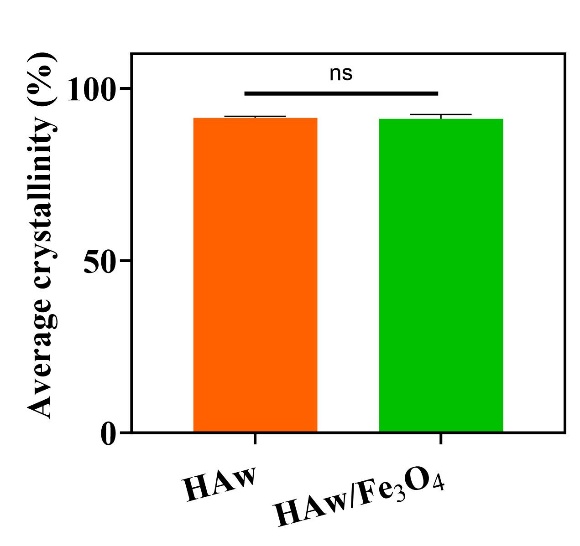


**Figure S1.** The average crystallinity of HAw and HAw/Fe_3_O_4_. ns, no significance, *n* = 3.

**Table S3** Content of the material phase in HAw/Fe_3_O_4._

|  | HAw | Fe_3_O_4_ |
| --- | --- | --- |
| Wt% | 8.7% | 91.3% |


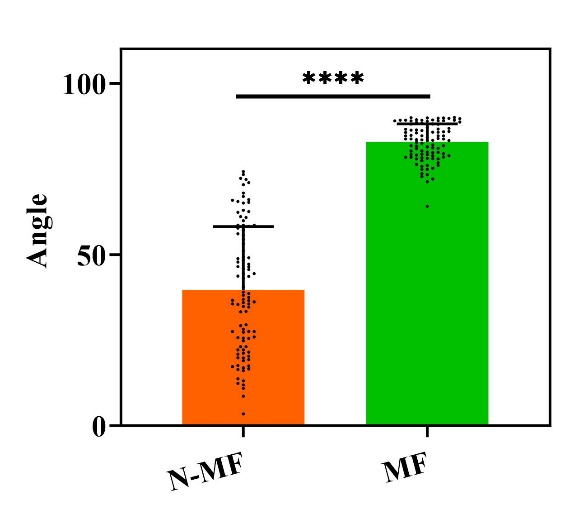


**Figure S2.** Angle between HAw/Fe_3_O_4_ and the horizontal direction in the presence and absence of magnetic field. **** *p* < 0.0001.


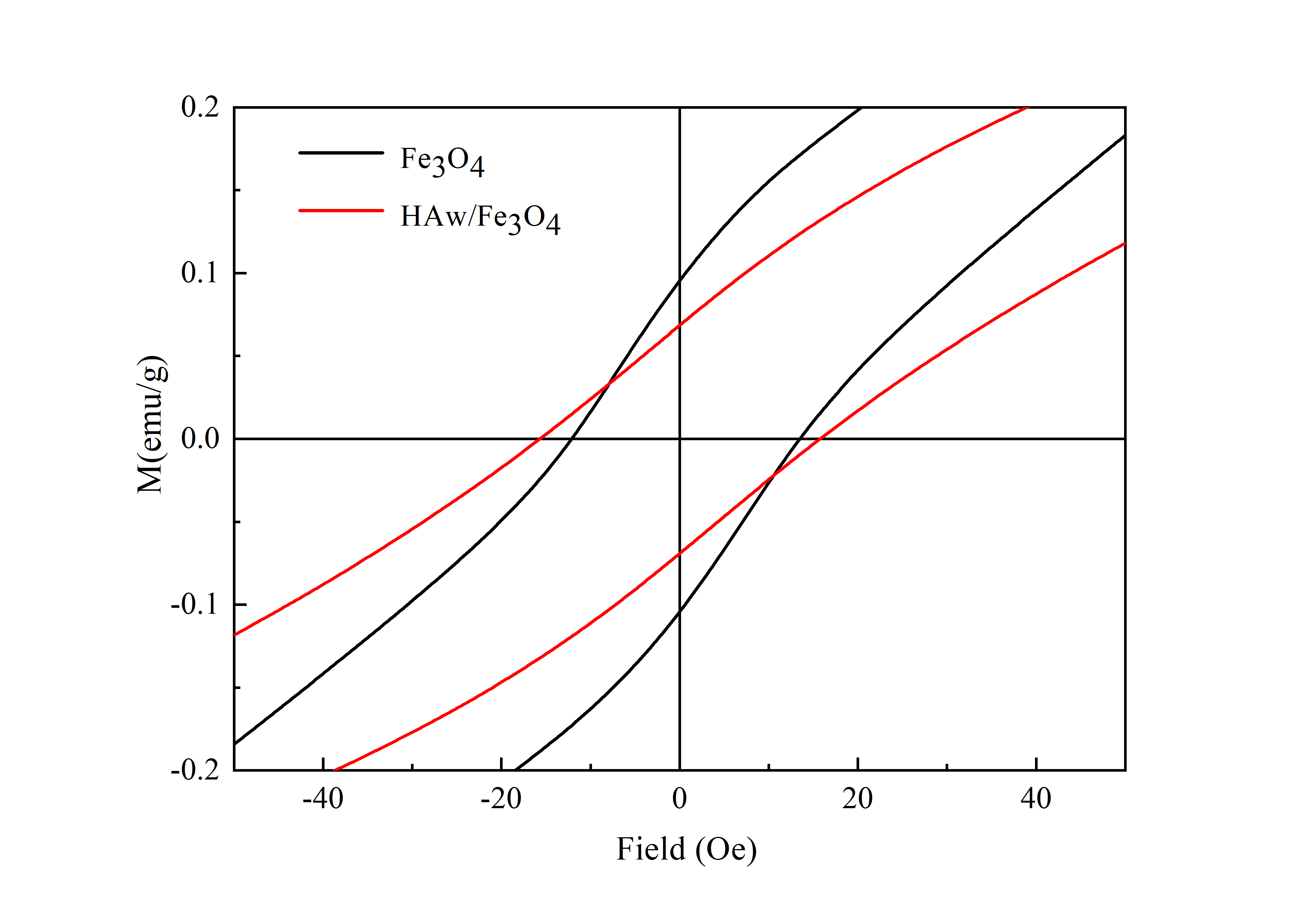


**Figure S3**. Coercive forces curves of Fe_3_O_4_ and HAw/Fe_3_O_4_.


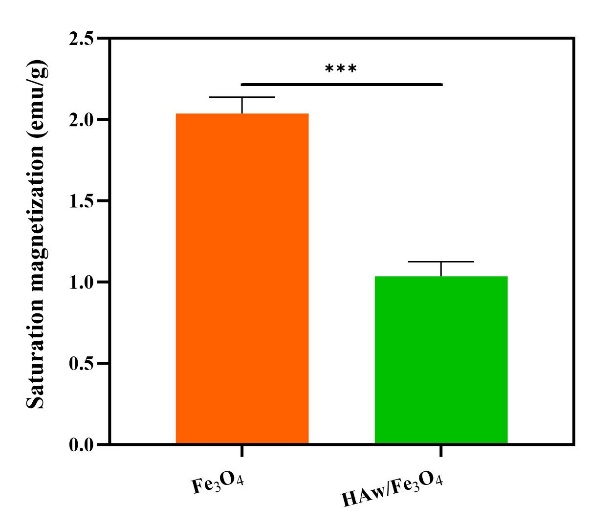


**Figure S4.** Saturation magnetization intensities of Fe_3_O_4_ and HAw/Fe_3_O_4_. *** *p* < 0.001
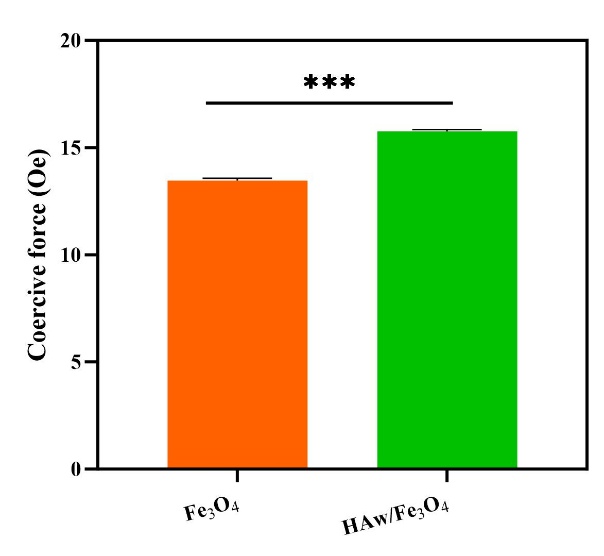


**Figure S5.** Coercive forces of Fe_3_O_4_ and HAw/Fe_3_O_4_. *** *p* < 0.001.

**Table S4.** Primer sequences for GAPDH, ALP, Runx2, Col-I and OCN

| Gene | Primer sequence | Length (bp) | Size (bp) |
| --- | --- | --- | --- |
| GAPDH | F5'-CTGGAGAAACCTGCCAAGTATG | 22 | 138 |
|  | R5'-GGTGGAAGAATGGGAGTTGCT | 21 |  |
| ALP | F5'-CGGCACCTGCCTTACCAACT | 20 | 171 |
|  | R5'-ACTGTGGAGACGCCCATACC | 20 |  |
| Runx2 | F5'-TACCCAGGCGTATTTCAGATGAT | 23 | 198 |
|  | R5'-TGTAAGTGAAGGTGGCTGGATAGT | 24 |  |
| Col-I | F5'-CCCAGCGGTGGTTATGACTT | 20 | 257 |
|  | R5'-TCGATCCAGTACTCTCCGCT | 20 |  |
| OCN | F5'-GAGCTCAACCCCAATTGTGAC | 21 | 81 |
|  | R5'-AACGGTGGTGCCATAGATGC | 20 |  |


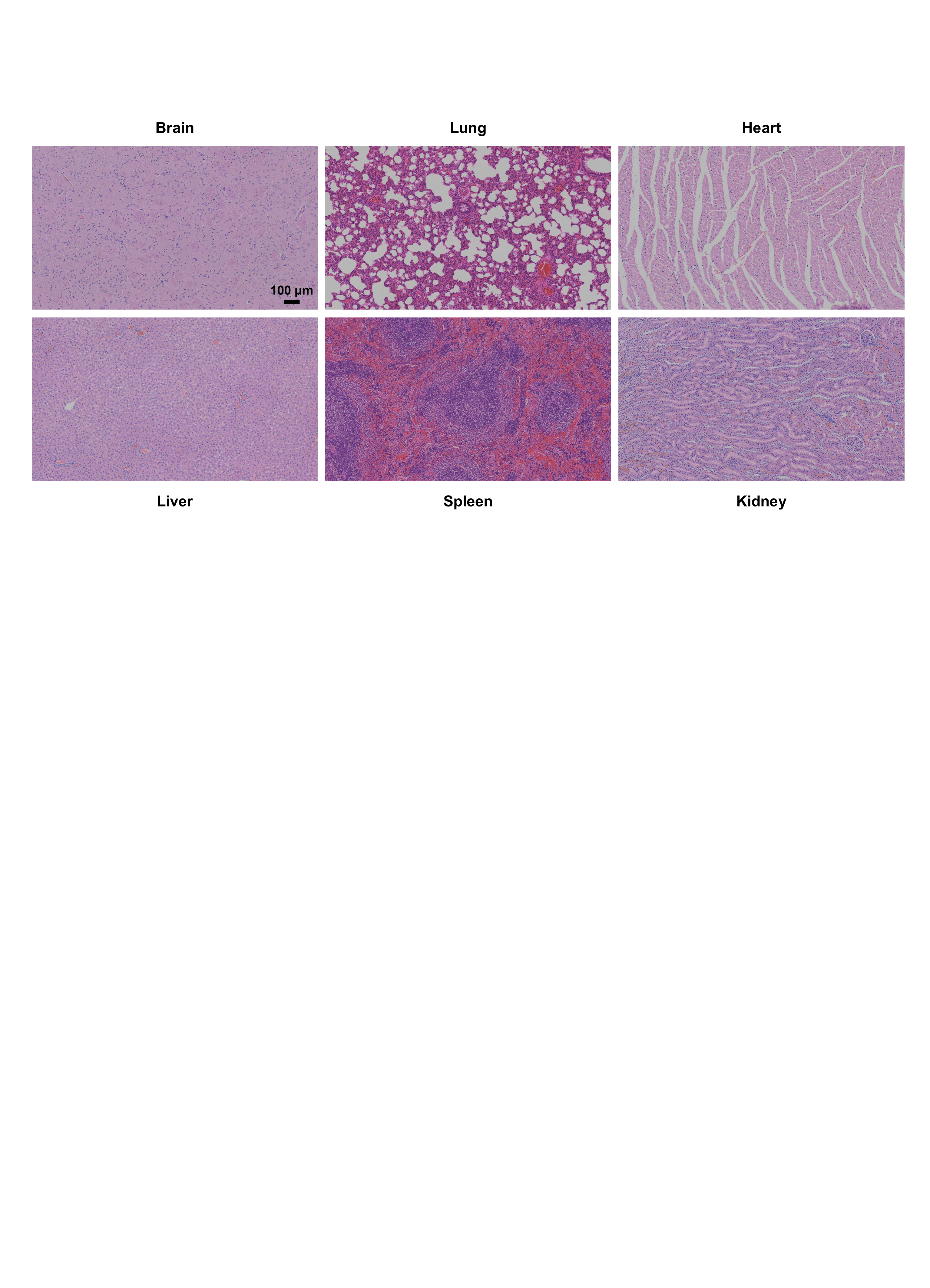


**Figure S6.** H&E staining of rat brain, lung, heart, liver, spleen and kidney sections after the magnetic HAw/Fe_3_O_4_ implantation in femur defects.
